# Dithering suppresses half-harmonic neural synchronisation to photic stimulation in humans

**DOI:** 10.1016/j.brs.2026.103111

**Published:** 2026

**Authors:** Benoit Duchet, Samini Subramaniam, Alexander Greenway, Shenghong He, Nicholas Shackle, Alek Pogosyan, Timothy Denison, Andrew Sharott, Huiling Tan, Rafal Bogacz

**Affiliations:** a Brain Network Dynamics Unit, Nuffield Department of Clinical Neuroscience, University of Oxford, Oxford, United Kingdom; b MRC Centre of Research Excellence in Restorative Neural Dynamics, United Kingdom; c University of Oxford, Oxford, United Kingdom; d Queen Mary University of London, London, United Kingdom; e Institute of Biomedical Engineering, Department of Engineering Sciences, University of Oxford, Oxford, United Kingdom

**Keywords:** Half-harmonic entrainment, Subharmonic entrainment, Photic stimulation, Dithered stimulation, EEG, Deep brain stimulation

## Abstract

**Background:**

While entraining neural rhythms using brain stimulation has been suggested as a therapeutic mechanism to normalise brain activity in conditions such as depression, chronic pain, or Alzheimer’s disease, periodic stimulation can also inadvertently entrain brain rhythms at sub- and superharmonics of the stimulation frequency, which could lead to deleterious effects. Slightly jittering stimulation pulses (called “dithering”) was previously proposed on the basis of mathematical modelling to selectively entrain a target neural rhythm while avoiding harmonic entrainment. In this study, we investigated the potential of dithering in humans.

**Methods:**

We recorded EEG in healthy adults during photic stimulation (light flicker) under periodic, dithered, reduced-strength, and control conditions. Synchronisation was quantified using spectral power and the phase-locking value.

**Results:**

We showed that dithering suppresses half-harmonic synchronisation relative to perfectly periodic flicker, and that dithering affects synchronisation at the stimulation frequency less than at the half-harmonic. This was also the case for a periodic condition with reduced stimulation strength, as predicted by theory. Furthermore, we demonstrated using synthetic data and modelling that the half-harmonic responses observed in participants cannot be explained by the superposition of evoked responses (even when modulated at the half-harmonic frequency), and are better matched by a minimal oscillator model.

**Conclusion:**

Our findings are consistent with half-harmonic EEG synchronisation in response to photic stimulation predominantly reflecting half-harmonic entrainment rather than the summation of evoked responses, and with dithering being an effective strategy to suppress subharmonic entrainment without reducing the energy delivered.

## Highlights

• Dithering can suppress 1:2 synchronisation to photic stimulation in humans.
• This is done without reducing the energy delivered.
• These 1:2 responses are inconsistent with the superposition hypothesis.
• These 1:2 responses are better matched by neural entrainment.

## Introduction

Entraining neural rhythms using brain stimulation has been suggested as a new therapeutic mechanism to normalise brain activity. For example, emerging approaches target the alpha band (8–12 Hz) using transcranial or sensory stimulation in individuals with depression or chronic pain [1], [2], [3], [4]. Gamma band (30–100 Hz) entrainment also hold promises, using sensory stimulation in Alzheimer’s disease [5], [6], [7], [8], and transcranial alternating current stimulation as well as deep brain stimulation (DBS) in Parkinson’s disease (PD) [9], [10].

However, periodic stimulation can also entrain neuronal rhythms at subharmonics and superharmonics of the stimulation frequency, which could lead to harmful effects. For example, finely-tuned gamma oscillations can be entrained at half the frequency of DBS in patients with PD [11], [12], [13], [14], [15] and dystonia [16]. This subharmonic entrainment was initially thought to promote debilitating involuntary movements known as dyskinesia [11], [12]. While recent studies have uncovered a more complex relationship between this subharmonic entrainment and dyskinesia [15], [17], they support the general principle that unintended entrainment can functionally disconnect neural oscillations, which could in some cases lead to unintended behavioural manifestations. Following this principle, DBS frequency was set to avoid subharmonic entrainment of rhythms associated with epileptic seizures in a canine with epilepsy [18].

**Fig. 1:**
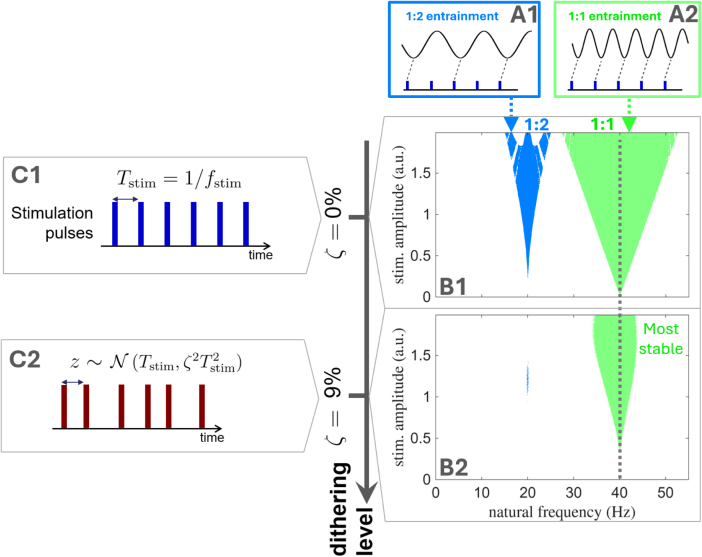
**Predicted effect of dithering on subharmonic entrainment.** When stimulation is perfectly periodic as depicted in **C1** ($f_{\text{stim}}$ denotes the stimulation frequency), neural oscillators may be entrained at the stimulation frequency but also at the half-harmonic of the stimulation frequency (as well as superharmonics and other subharmonic ratios, not shown here). Corresponding entrainment regions (called Arnold tongues) are represented in **B1** for uncoupled neural oscillators modelled in [19]. Stimulation is provided at 40 Hz (grey dashed line), with stimulation amplitude shown on the vertical axis, and the natural frequency of oscillators on the horizontal axis. Entrainment to the stimulation frequency (1:1 entrainment) is observed in the green region, while half-harmonic entrainment (1:2 entrainment) is observed in the blue region. Schematics representing both types of entrainment are shown in **A1–2**. With dithering, stimulation pulses are slightly jittered (**C2**), and past a certain dithering level (level of noise in the stimulation period, denoted by ζ), only the 1:1 Arnold tongue subsists (green tongue in **B2**).

To selectively entrain a target neural rhythm while avoiding potential harmful effects from sub- and superharmonic entrainment, a stimulation approach called “dithering” has been proposed [19]. In its simplest form, dithering involves jittering stimulation pulses, such that the duration of each inter-pulse interval varies slightly from the mean stimulation period. Since sub- and superharmonic entrainment are less stable than entrainment at the mean stimulation frequency, a level of dithering can be found that preserves entrainment at the target frequency while suppressing sub- and superharmonic entrainment (Fig. 1). The ability of dithered stimulation to achieve selective entrainment was established theoretically and verified computationally in networks of coupled neural oscillators [19], but has not yet been tested experimentally.

Figure adapted from [19].

Here, we test the ability of dithering to modulate subharmonic synchronisation in healthy humans using photic stimulation (light flicker) and electroencephalography (EEG). The ongoing debate on whether photic stimulation entrains neural activity at the stimulation frequency (with neural oscillators synchronising to the stimulation frequency) or simply evokes time-locked neural responses to each individual flash [20], [21], [22], [23], [24] precludes us from concluding on the impact of dithering on entrainment at the stimulation frequency and its superharmonics. Instead we consider subharmonic responses to photic stimulation, which have been reported in numerous human studies [6], [25], [26], [27], [28], [29]. We show that these subharmonic responses can be suppressed by dithered stimulation, and present evidence that these subharmonic responses are not consistent with evoked responses and likely reflect subharmonic entrainment.

**Fig. 2:**
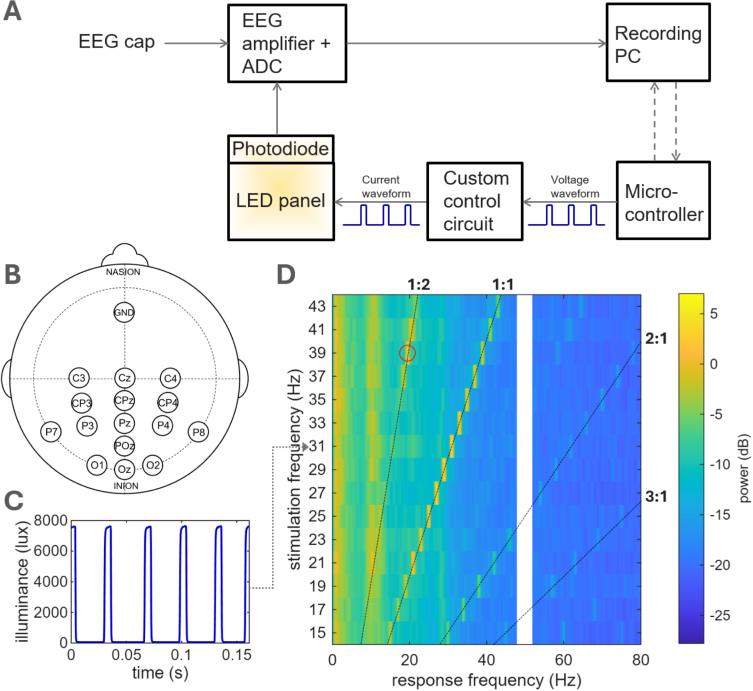
**Measuring EEG responses to photic stimulation. A**: Sketch of the experimental setup. Dashed arrows indicate intermittent communication between devices. **B**: EEG electrodes used in the study. **C**: Example photodiode output during 31 Hz periodic stimulation (corrected using the calibration presented in Fig S.1). **D**: EEG responses to periodic stimulation (full modulation depth) averaged over channels for stimulation frequencies between 15 and 43 Hz in one participant. The colorbar shows EEG power averaged over channels, such that each row of this plot represents the EEG power spectrum for the corresponding stimulation frequency on the vertical axis. The response at 50 Hz is hidden due to line noise. The alpha rhythm is visible around a response frequency of 10 Hz. Dashed black lines highlight responses at integer ratios of the stimulation frequency. The maximum response at half the stimulation frequency is obtained for a stimulation frequency of $f_{\text{max 1:2}}=39\;\mathrm{Hz}$ and is indicated by a red circle.

## Methods

### Participants

We recruited right-handed healthy participants, who were screened to minimise the risk of photic stimulation causing epileptic seizures. We included participants above 20 years old (to reduce risk of undiagnosed epilepsy) and below 60 years old, see Table A in the supplementary material for detailed inclusion/exclusion criteria. The study was approved by the Medical Sciences Interdivisional Research Ethics Committee of the University of Oxford (approval reference: R93268/RE001), and all participants gave written informed consent. We recorded data from 16 participants (8 female, mean age = 31.6±10.0 years old, see Table 1).

### Photic stimulation

Photic stimulation was delivered via a white light emitting diode (LED) strip with a diffuser in front. The LED strip was driven by a Teensy 4.1 microcontroller, allowing precise control of the stimulation waveform. The microcontroller’s voltage output was converted into a current waveform using a custom control board (Fig. 2A). Participants were sat approximately 75 cm away from the photic stimulation device with the device at eye level. The illuminance at the level of the participants’ eyes was approximately 70 lux (during continuous illumination, measured using an RS-92 light meter, RS PRO). The light flicker was presented as square pulses (20% duty cycle) since square pulses evoke a stronger response at the stimulation frequency than sinusoidal waveforms [30], and were also found in pilot testing to elicit a stronger subharmonic response. Sinusoidal waveforms did not elicit strong enough 1:2 responses across participants to allow us to test the efficacy of dithering.

### Recorded signals

We recorded EEG using a TMSi (Twente Medical Systems) amplifier and a 10–10 EEG cap with 64 electrodes. Only 15 electrodes were used (see Fig. 2B), located more posteriorly towards the visual cortex and referenced using common average referencing (based on the 15-electrode subset). The AFz electrode was used as a ground electrode. Impedances were kept under 5 kΩ. EEG data were recorded with a sampling rate of 4096 Hz.

A photodiode placed on the LED panel allowed synchronised acquisition of the illumination waveform and EEG signal. The non-linearity of the photodiode was corrected using a calibration procedure (see Fig S.1 in the supplementary material for more details).

### Experimental paradigm

**Fig. 3:**
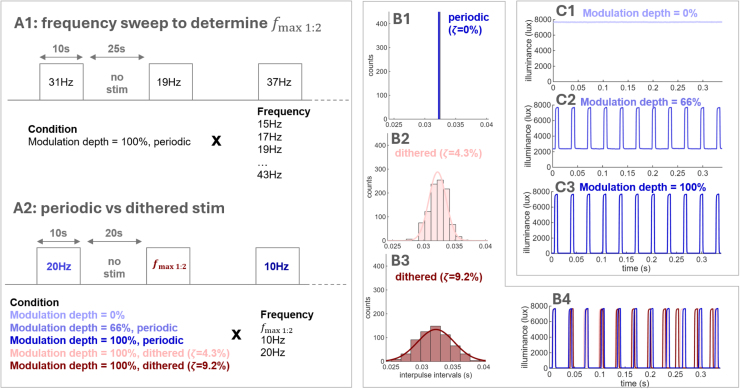
**Experimental paradigm and stimulation conditions. A1**: Schematic of the frequency sweep protocol used to determine $f_{\text{max 1:2}}$. **A2**: Schematic of the protocol used to compare dithered stimulation to periodic stimulation. **B**: Distribution of inter-pulse intervals for the periodic (B1) and dithered conditions (B2–B3). The dithering level ζ scales the standard deviation of the inter-pulse interval distribution. B4 shows example stimulation trains for the three conditions. **C**: Stimulation trains with different modulation depths. The modulation depth inversely scales the “off” part of the stimulation pulse. A modulation depth of 0% corresponds to continuous illumination (control condition, C1). Examples in panels B-C are from one participant with a stimulation frequency of 31 Hz.

The experimental protocol consisted of two parts. In the first part, we identified the stimulation frequency leading to the largest power response at the half-harmonic (denoted $f_{\text{max 1:2}}$). Participants were exposed to a frequency sweep consisting of 10 s trials with periodic flicker (ζ=0%) from 15 Hz to 43 Hz in 2 Hz increments (Fig. 3A1), with two repeats per frequency. This range was previously found to elicit half-harmonic responses in most subjects [28]. Modulation depth, defined as $\left(I_{\text{max}}-I_{\text{min}}\right)/I_{\text{max}}$ where $I_{\text{max}}$ is the illuminance during the “on” part of the pulse, and $I_{\text{min}}$ is the illuminance during the “off” part of the pulse (see Fig. 3C) , was 100%. The stimulation frequency with the largest averaged power response (obtained as detailed in the next section) at the half-harmonic of the stimulation frequency was identified as $f_{\text{max 1:2}}$ (see example in Fig. 2D).

In the second part, periodic stimulation was compared to dithered stimulation by exposing participants to 10 s trials with flicker of frequency $f_{\text{max 1:2}}$, but also 10, and 20 Hz. Dithering was implemented by sampling the time interval from one stimulation pulse to the next from a normal distribution $N\left(T_{\text{stim}},\zeta^{2}T_{\text{stim}}^{2}\right)$, where $T_{\text{stim}}=1/f_{\text{stim}}$, with $f_{\text{stim}}$ the target stimulation frequency, and ζ the dithering level (Fig. 3B). For each stimulation frequency, periodic conditions (ζ=0%) with modulation depths of 0% (control condition with no flicker), 66%, and 100% were included, as well as two dithered conditions (ζ=4.3% and ζ=9.2%, with a modulation depth of 100% in each case) – see Fig. 3A2. Each stimulation condition was repeated four times. The manuscript focuses on stimulation at $f_{\text{max 1:2}}$, but results for 10 Hz and 20 Hz stimulation are included in the supplementary material.

During both experimental parts, the order in which stimulation conditions (including repeats) were presented was randomised. Stimulation periods lasted 10 s and were followed by 20 s rest periods (25 s during the frequency sweep). A stimulation duration of 10 s was chosen as a compromise to obtain enough data for robust quantification of (possibly transient) phase-synchronisation while minimising participant fatigue.

### Quantifying synchronisation

Synchronisation between EEG activity and stimulation was quantified using two complementary measures: spectral power and the phase-locking value (PLV). While spectral power provided a coarse measure of frequency-specific response, we used the PLV to obtain a more direct measure of phase synchronisation between the EEG signal and stimulation. To increase sensitivity to transient locking, we also computed a windowed version of the PLV.

#### Artefact rejection and trimming

All trials were visually inspected for artefacts by a rater who was blinded to the trial conditions, and trials with clear, large artefacts such as motion, muscle, or electrode pop artefacts were not included in the analysis. Rejection rates were low and did not show a pronounced systematic bias across stimulation conditions (see Section C in the supplementary material for more details). We trimmed 200 ms off the beginning and the end of each trial to remove potential onset and offset effects.

#### Power estimation

A coarse assessment of synchronisation was first performed by computing the power of the EEG response. The power of the EEG response was obtained for each stimulation frequency using Welch’s method (8 segments per trial with 50% overlap, i.e. segments of duration 2.2 s), and averaged across trials and EEG channels. During the frequency-sweep experiment, these power estimates were used to identify $f_{\text{max 1:2}}$ for each participant. In the main experiment, power was used as a coarse assessment of the response at the stimulation frequency and its half-harmonic.

#### PLV computation

To better quantify the degree of EEG signal synchronisation to stimulation, we computed the phase-locking value (PLV), which measures the concentration of the EEG signal’s phase according to the timing of stimulation. We considered the PLV at integer ratios of the stimulation frequency (1:1 and 1:2 in the main text, as well as 2:1 and 3:1 in the supplementary material). In general, synchronisation at the n:m ratio corresponds to m stimulation pulses spanning n cycles of the brain signal. For each n:m ratio considered, the EEG data was bandpass filtered around $f_{\text{filt}}=\left(n/m\right)f_{\text{stim}}$, with a half-width relative to the filter’s center frequency given by $df=\left(n/m\right)f_{\text{stim}}/10$, corresponding to df=1Hz for $f_{\text{stim}}=10\;\mathrm{Hz}$ at 1:1. We applied a second order butterworth filter both forwards and backwards to minimise phase distortion, and obtained the Hilbert phase $\psi_{\text{n:m}}$ for each n:m ratio considered. For each trial, and each EEG channel, the PLV was computed for n:1 ratios as

(1) $${\text{PLV}}_{\text{n:1}}^{\text{data}}=\frac{1}{N}\left|\overset{N}{\underset{k=1}{\sum }}e^{i\psi_{\text{n:1}}\left(t_{k}\right)}\right|,$$

where N is the number of pulses in the trial considered, $\psi_{\text{n:1}}\left(t\right)$ is the Hilbert phase obtained from the corresponding filtered EEG signal, and the $t_{k}$’s are the times of the PLV triggers, which are described below. The PLV was also computed for 1:2 by considering only every other trigger using

(2) $${\text{PLV}}_{\text{1:2}}^{\text{data}}=\frac{1}{\left\lfloor N/2\right\rfloor }\left|\overset{\left\lfloor N/2\right\rfloor }{\underset{k=1}{\sum }}e^{i\psi_{\text{1:2}}\left(t_{2k}\right)}\right|,$$

with ⌊.⌋ the floor function. PLV values were averaged over channels and non-rejected trials, with averaged values denoted ${\bar{\text{PLV}}}_{\text{n:m}}^{\text{data}}$.

We computed the PLV using two types of PLV triggers, which are the same for periodic stimulation but not for dithered stimulation (Fig. 5A). The first type, which we call “fixed PLV triggers”, was used to assess synchronisation at the average stimulation frequency, and employed as PLV triggers the times of stimulation in the absence of dithering. For ζ=0%, we used the times of stimulation from the current trial, whereas for ζ>0%, we used the times of stimulation from the first non-rejected trial with the same stimulation frequency and modulation depth but with ζ=0%. The second type, which we call “dithered PLV triggers”, was used to contrast the data with various models, and employed as PLV triggers the times of stimulation in the current trial regardless of ζ. In both cases, the times of stimulation were identified through threshold crossings of pulses’ leading edges in the illumination signal obtained from the photodiode.

In all cases, to remove from PLV estimates the contribution of spurious phase-locking due to phase alignment with stimulation happening by chance, we computed the PLV for pink noise signals put through the exact same analysis pipeline as the data, including filtering (using the same PLV triggers used for the data). The PLV estimate with the noise contribution removed was obtained as

(3) $${\text{PLV}}_{\text{n:m}}={\bar{\text{PLV}}}_{\text{n:m}}^{\text{data}}-{\bar{\text{PLV}}}_{\text{n:m}}^{\text{noise}},$$

where ${\bar{\text{PLV}}}_{\text{n:m}}^{\text{noise}}$ is the PLV for noise computed as for the data (using Eqs. (1) or (2)), averaged as for the data.

To increase sensitivity to transient locking which may be present in the data, we also computed a windowed version of the PLV, whereby we obtained the PLV in windows of duration relative to the filter’s center frequency given by $500\times 40/f_{\text{filt}}\;\mathrm{ms}$ (500 ms for $f_{\text{filt}}=40\;\mathrm{Hz}$), with overlap also relative to the filter’s center frequency given by $200\times 40/f_{\text{filt}}\;\mathrm{ms}$ (200 ms for $f_{\text{filt}}=40\;\mathrm{Hz}$), and averaged the resulting values. We denote the windowed PLV by ${\text{PLV}}_{\text{n:m}}^{\text{win}}$ (with noise removed as per Eq. (3)).

#### Exclusion of participants from the analysis and statistical tests

We excluded from the analysis five participants who had a very weak response at the half-harmonic of stimulation (${\text{PLV}}_{\text{1:2}}^{\text{win}}<0.1$) in the periodic condition for $f_{\text{stim}}=f_{\text{max 1:2}}$ and a modulation depth of 100%. We also excluded from the analysis one participant who did not have at least two non-rejected trials for each stimulation condition for $f_{\text{stim}}=f_{\text{max 1:2}}$.

To compare synchronisation between conditions as well as between empirical data and synthetic data at the group level, we performed signed rank tests for paired data (n = 10 pairs for all tests). All statistical tests in this study were performed under false discovery rate (FDR) control at 5% according to the Benjamini and Hochberg procedure [31]. All p-values < 0.05 were found to also be significant under FDR control.

### Synthetic data based on the superposition of evoked potentials hypothesis

To assess whether the PLVs observed in data at the 1:2 subharmonic could be accounted for simply by the summation of sensory evoked potentials with ongoing neural activity, synthetic data were generated and put through the same analysis pipeline as the data. For each participant included in the analysis, we generated synthetic data by 1) replacing each stimulation trial by EEG data corresponding to one of the participant’s randomly selected control trials (where the LED panel was on but not flashing), and 2) aligned to each stimulation trigger recorded from the photodiode in the stimulation trial, summing scaled averaged evoked potentials to the control EEG data (Fig. 5B). Averaged evoked potentials were obtained by averaging all periods ($1/f_{\text{stim}}$ long epochs) directly following stimulation triggers in trials at the stimulation frequency considered, with ζ=0%, and a modulation depth of 100%. This was done independently for each EEG channel and each subject, using the corresponding EEG data high-passed at 1 Hz, and low-passed at 80 Hz, with a notch filter at 50 Hz (line noise in the UK). Other types of averaged evoked potentials were also considered as described in Section A in the supplementary material, namely the averaged flash visual evoked potential (VEP) obtained in one participant, and averaged evoked potentials including frequency components at half the stimulation frequency. The scale factor S was chosen to match the channel-average PLV at the stimulation frequency, ζ=0%, and 100% modulation depth in each participant’s data and synthetic data.

We also considered the possibility that sensory evoked potentials could be modulated at half the stimulation frequency by non-linear sensory mechanisms such as saturation or gain control [32], [33]. For example, the gain of the response could be reduced for a short period after a strong flash. To account for such a potential mechanism, we alternated between modulating consecutive evoked potentials by the factor $1+m_{\text{1:2}}$, and the factor $1-m_{\text{1:2}}$ (Fig. 5B). This is a general approach to introduce period-doubling (1:2 modulation) in the response gain. It does not specify any particular underlying mechanism but can describe the period-doubling response of any autonomous (time-invariant) non-linear system (see examples of non-linear saturation model and non-linear feedback model in Fig S.2 and Section D in the supplementary material). To show that time dependence of $m_{\text{1:2}}$ is unlikely to change the results of our analyses, we also considered a linear decrease of $m_{\text{1:2}}$ over the course of each stimulation block, and a slow sinusoidal fluctuation of $m_{\text{1:2}}$ over the course of each stimulation block (four cycles per stimulation block), see Fig S.3A1-2 in the supplementary material. In all cases, we chose $m_{\text{1:2}}$ to match the channel-average PLV at half the stimulation frequency, ζ=0%, and 100% modulation depth in each participant’s data and synthetic data. Additionally, to ensure that using averaged responses did not introduce biases due to the lack of within-trial and between-trial variability, we constructed synthetic data using randomly sampled single-pulse, single-trial responses instead of using averaged evoked responses (Fig S.4A1-4, C1-4 in the supplementary material).

### Oscillator model

To investigate whether the PLV levels observed in empirical data at the 1:2 subharmonic may be better accounted for by entrainment of an oscillator, we simulated a simple oscillator model. As in [19], we considered the sine circle map, which is the simplest model describing the influence of periodic stimulation on a single neural oscillator. Entrainment can arise because a stimulus may advance or delay the phase of an oscillator depending on the phase at which it is applied. This concept is captured by the phase response curve (PRC) of the oscillator, which describes the change in phase of the oscillator as a function of the stimulation phase. The PRC of the sine circle map is a simple sinusoid (Z(θ)=sinθ). The model we used maps the phase of an oscillator right before stimulation pulse n (denoted $\theta_{n}$) to the phase of the oscillator right before stimulation pulse n+1 (denoted $\theta_{n+1}$). The map is described by

(4) $$\theta_{n+1}=\theta_{n}+2\pi \frac{T_{\text{stim}}\left(1+z_{n}\right)}{T_{0}\left(1+y_{n}\right)}+I\sin\theta_{n},$$

where I is the stimulation magnitude and, to model dithered stimulation, the stimulation period $T_{\text{stim}}=1/f_{\text{stim}}$ is multiplied by $\left(1+z_{n}\right)$, with $z_{n}$ normal random numbers sampled from $N\left(0,\zeta^{2}\right)$, and ζ the dithering level. We also added noise to the oscillator’s natural period $T_{0}$ to allow us to reduce the PLV at the 1:2 subharmonic in the absence of dithering to the average value observed in the data. The oscillator’s noise level is denoted $\zeta_{\text{mod}}$, and $y_{n}$ are normal random numbers sampled from $N\left(0,\zeta_{\text{mod}}^{2}\right)$.

For comparison with empirical data group average results, we picked $f_{\text{stim}}=30\;\mathrm{Hz}$ (close to the group average of $f_{\text{max 1:2}}$). We matched the PLV at the 1:2 subharmonic in the absence of dithering to the average value observed in the data by using $\zeta_{\text{mod}}=0.083$. For each dithering level used in the data, we simulated 40 trials of 6000 pulses each. To calculate the PLV using fixed PLV triggers, we sampled the phase at dt=0.0001 s between points given by the map (assuming the oscillator’s frequency stays constant between stimulation pulses). Because the ${\text{PLV}}_{\text{1:2}}$ metric is also sensitive to PLV at the 1:1 ratio in the absence of filtering (which, contrary to the data, cannot be applied here since the oscillator only has a phase and no amplitude), a ${\text{PLV}}_{\text{1:2}}$ metric comparable to the data was obtained by subtracting ${\text{PLV}}_{\text{1:1}}$ to ${\text{PLV}}_{\text{1:2}}$ (both computed at the same oscillator natural frequency and amplitude). This process also removes spurious synchronisation due to chance. We averaged these results within a region of interest focused on the center of the 1:2 tongue (natural frequency 15 Hz, and stimulation magnitude between 0.85 and 1.35 a.u.).

## Results

Since there is individual variability in which stimulation frequencies lead to subharmonic responses to photic stimulation, we first identified for each participant the stimulation frequency giving rise to the largest power response at the half-harmonic (denoted $f_{\text{max 1:2}}$) using a frequency sweep from 15 to 43 Hz (Fig. 3A1). For the included datasets, the mean $f_{\text{max 1:2}}$ was 32.4±6.7Hz (individual values reported in Table 1, also see example response to stimulation frequency sweep in Fig. 2D). The half-harmonic of stimulation was in the alpha band for only two participants, with the half-harmonic falling in the beta band (13–35 Hz) for all other participants. The variability of $f_{\text{max 1:2}}$ across both repeats in included participants was on average 2.2 Hz (Fig S.5 in the supplementary material).

We next assessed the effect of dithering on half-harmonic synchronisation (Fig. 3A2), and used synthetic data and modelling to characterise the nature of this half-harmonic synchronisation. Following previous model predictions [19] (Fig. 1B2), we considered the dithering levels ζ=9.2% (predicted to be sufficient for the 1:2 Arnold tongue to disappear), ζ=4.3% (an intermediate level), and ζ=0% (the periodic case) with the same overall energy delivered (same modulation depth of 100%) - see Fig. 3B. Since theory also predicts that sufficiently reducing stimulation amplitude will suppress 1:2 entrainment (the system will leave the 1:2 Arnold tongue, see Fig. 1B1), a periodic condition with a reduced modulation depth of 66% was also included (Fig. 3C2). Finally, we included a control condition (modulation depth of 0%, i.e. continuous illumination without flicker, Fig. 3C1).

**Table 1:** Participant demographics and inclusion for analysis based on EEG response.

Participant ID	Gender	Age	$f_{\text{max}1:2}$ (Hz)	${\text{PLV}}_{\text{1:2}}^{\text{win}}$	Included in analysis
1	F	21	31	0.255	yes
2	F	21	43	0.081	no
3	M	52	31	0.145	yes
4	F	35	43	0.021	no
5	M	29	43	0.216	yes
6	F	22	37	0.026	no
7	M	51	39	NA	no
8	M	41	39	0.024	no
9	F	20	39	0.113	yes
10	M	38	39	0.137	yes
11	M	31	19	0.102	yes
12	F	28	33	0.133	yes
13	M	28	29	0.184	yes
14	M	35	31	0.182	yes
15	F	23	33	0.036	no
16	F	31	29	0.182	yes

Out of 16 recorded datasets, 10 were included in the study. The fourth column gives the stimulation frequency identified during the initial frequency sweep as giving rise to the largest power response at the half-harmonic frequency ($f_{\text{max 1:2}}$). The fifth column gives ${\text{PLV}}_{\text{1:2}}^{\text{win}}$ for $f_{\text{stim}}=f_{\text{max 1:2}}$, ζ=0%, and a modulation depth of 100%. The ${\text{PLV}}_{\text{1:2}}^{\text{win}}$ entry for participant 7 reads NA (not available) because all trials were rejected for $f_{\text{stim}}=f_{\text{max 1:2}}$, ζ=0%, and a modulation depth of 100%.

After data recording, we excluded six datasets from further analysis due to the lack of subharmonic response to periodic stimulation or too many rejected trials (see detailed rejection criteria in Section “Quantifying synchronisation”, resulting in 10 participants included for analysis.

### Dithering suppresses half-harmonic power

**Fig. 4:**
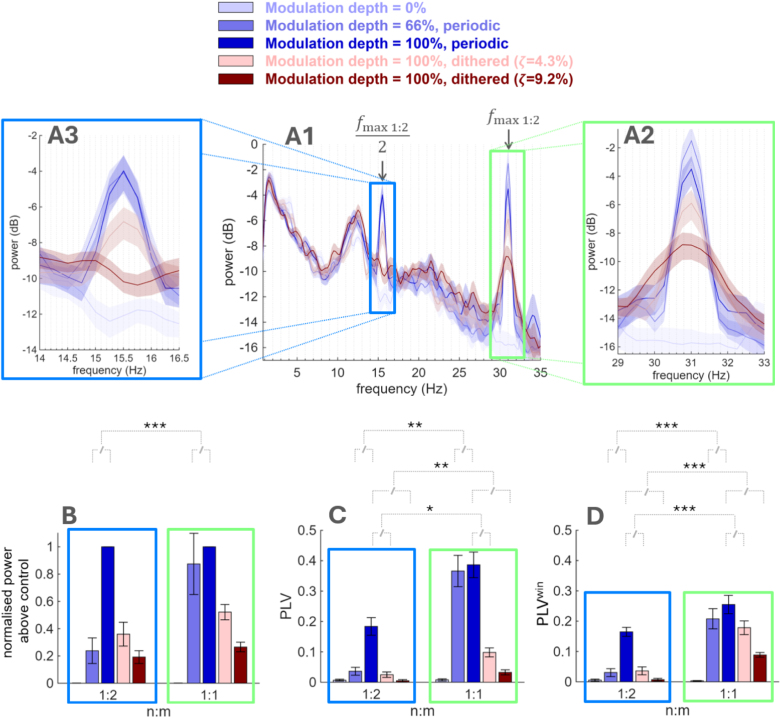
**Dithering suppresses half-harmonic synchronisation. A1**: Power spectrum of participant 14 (averaged over trials and EEG channels) in response to periodic (shades of blue) and dithered (shades of red) photic stimulation at $f_{\text{max 1:2}}=31\;\mathrm{Hz}$. The lightest shade of blue corresponds to a control condition (continuous illumination, no flicker). **A2** and **A3** are zoomed-in inserts centered on the stimulation frequency and its half-harmonic, respectively. **B**: Normalised power above control at the stimulation frequency (1:1) and its half-harmonic (1:2) at the group level (the value for the periodic condition with 100% modulation depth is one by definition of the normalisation). **C**: PLV at the stimulation frequency (1:1) and its half-harmonic (1:2) at the group level. **D**: Windowed PLV at the stimulation frequency (1:1) and its half-harmonic (1:2) at the group level. In panels B-D, n = 10, and significance is only indicated between ratios of conditions relative to the periodic condition with full modulation depth to avoid clutter. In panels C–D, the control condition has a PLV close to zero as expected (the noise contribution is removed in our PLV measure). All panels share the same legend. Error bars or shaded areas represent the standard error of the mean. *, **, and *** indicate p≤ 0.05, p≤ 0.01, and p≤ 0.001, respectively.

Dithering reduced the power of the half-harmonic response to photic stimulation compared to periodic stimulation at $f_{\text{max 1:2}}$. The power response of a representative participant is presented in Fig. 4A and shows a greater reduction in power at the half-harmonic for ζ=9.2% than for ζ=4.3%. We now perform all comparison between conditions at the group level across included participants. Because of between-participant differences in baseline power and maximum power response, we considered for group-level analysis the “normalised power above control”, defined as the difference between the power response for the condition of interest and the control condition, normalised by the difference between the power response of the periodic condition with full modulation depth (which elicits the strongest response) and the control condition. As seen in Fig. 4B, both dithering levels (as well as the reduced modulation depth condition) significantly reduced the normalised power above control at the half-harmonic (p<0.001 in all three cases, one-tailed). For both dithering levels, there was a trend in dithering reducing power at the half-harmonic of stimulation more than at the stimulation frequency (comparison of ratios relative to the periodic condition with full modulation depth at 1:1 and 1:2, p= 0.08 in both cases, one-tailed). The reduced modulation depth condition significantly decreased power at the half-harmonic of stimulation more than at the stimulation frequency (comparison of ratios relative to the periodic condition with full modulation depth at 1:1 and 1:2, p<0.001, one-tailed). Similar results were found when power is not normalised (see Fig S.6 in the supplementary material). Since the power of the response is a coarse measure of synchronisation with stimulation (increases in power do not always correlate with increases in synchronisation [34], [35]), we assessed synchronisation in a more specific manner using the PLV.

**Fig. 5:**
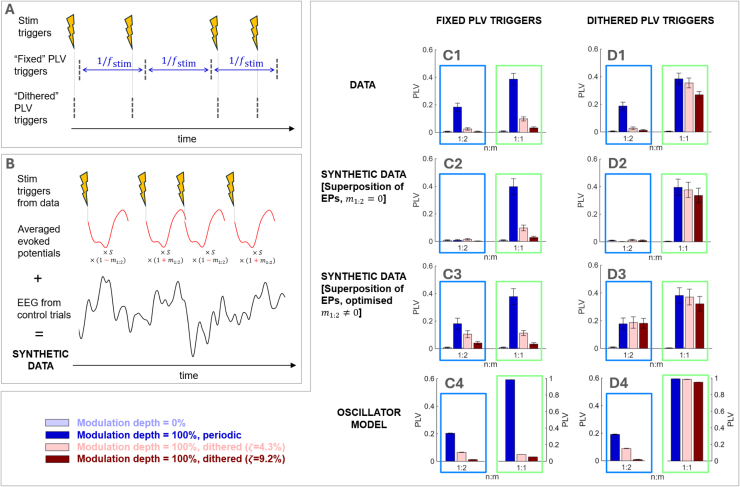
**Half-harmonic responses to photic stimulation are better explained by half-harmonic entrainment than the superposition of evoked responses. A**: Schematic of the difference between fixed PLV triggers and dithered PLV triggers ($f_{\text{stim}}$ is the stimulation frequency). **B**: Schematic illustrating how synthetic data is generated according to the superposition of evoked potentials hypothesis. For each participant, the scale factor S was determined to match the data ${\text{PLV}}_{\text{1:1}}$ for the periodic condition. In C3 and D3, the modulation factor $m_{\text{1:2}}$ was chosen to match the data ${\text{PLV}}_{\text{1:2}}$ for the periodic condition for each participant. **C–D**: PLV at the stimulation frequency (1:1) and its half-harmonic (1:2) at the group level, for fixed PLV triggers (C) and dithered PLV triggers (D). Comparison between the empirical data, synthetic data generated according to the superposition of evoked potentials hypothesis (without and with modulation at the half-frequency), and an oscillator model (sine circle map with noise). Panels C–D share the same legend, and error bars represent the standard error of the mean (error bars are hardly visible in C4 and D4 due to low cross-trial variability).

### Dithering suppresses half-harmonic PLV

Dithering strongly suppressed half-harmonic synchronisation to photic stimulation compared to periodic stimulation at $f_{\text{max 1:2}}$, even for the intermediate dithering level (Fig. 4C–D). At the group level, this effect was manifest both for a global PLV measure (Fig. 4C), where phase synchronisation is computed for 10 s trials, and a windowed PLV measure (Fig. 4D), which is also sensitive to transient phase synchronisation (in both cases: p<0.001 for both dithering levels, one-tailed). Moreover, synchronisation was reduced at the half-harmonic of stimulation more than at the stimulation frequency (comparison of ratios relative to the periodic condition with full modulation depth at 1:1 and 1:2): for the global PLV measure, p= 0.032 for ζ=4.3%, and p= 0.005 for ζ=9.2%, while for the windowed PLV, p= 0.001 for both dithering levels (one-tailed tests). The periodic condition with reduced modulation depth also showed a significant suppression of half-harmonic synchronisation, and a greater reduction in synchronisation at the half-harmonic of stimulation than at stimulation frequency as measured by both global and windowed PLVs (p≤ 0.002 for all four tests, one-tailed). While the periodic condition with reduced modulation depth better preserved synchronisation at the stimulation frequency as measured by the global PLV (Fig. 4C) than dithering, the intermediate dithering level preserved synchronisation at the stimulation frequency measured by the windowed PLV (Fig. 4D) similarly to the periodic condition with reduced modulation depth (p=0.38, two-tailed), and similarly suppressed 1:2 synchronisation (p=0.74, two-tailed). The global PLV measure for the intermediate dithering level was also above that of the control condition (noise level) at the stimulation frequency, p<0.001 (one-tailed), Fig. 4C. Differences between the global and windowed PLV measures are due to the sensitivity of the windowed measure to short but frequent periods of phase synchronisation (see Fig S.7 in the supplementary material). These are not captured by the global PLV since phase slips between these periods substantially lower the global measure. Averaged across conditions, within-subject PLV variability was lower than between-subject PLV variability, see Table B in the supplementary material for more details.

Our results were robust to changes in pre-processing choices as demonstrated by sensitivity analyses. Statistical significance achieved in Fig. 4C–D remained similar when: (1) doubling or halving the filter width (see Fig S.8A-B in the supplementary material); (2) doubling or halving the window duration used in the windowed PLV analysis (see Fig S.8C–D in the supplementary material); (3) using control data (modulation depth of 0%) instead of pink noise to remove the contribution of chance phase locking in PLV estimates (see Fig S.9 in the supplementary material); and (4) using a bipolar montage instead of common average referencing (see Fig S.10 in the supplementary material). We also show that small differences in trial rejection rates across conditions did not bias our results in Section C of the supplementary material (also see Fig S.11 in the supplementary material).

### Half-harmonic responses are better explained by half-harmonic entrainment than the superposition of evoked responses

To elucidate the nature of these half-harmonic responses, we generated synthetic data using averaged evoked potentials superimposed onto EEG control data according to the timing of stimulation in each trial and for each participant (Fig. 5B), and present results at the group level. The superposition of evoked potentials hypothesis could account for the PLV values obtained in the empirical data at the stimulation frequency (see 1:1 in Fig. 5C1-2 and D1-2) as well as superharmonics for 10 and 20 Hz stimulation (see Fig S.12 in the supplementary material). However, regardless of the type of PLV triggers used (see Fig. 5A), the linear superposition of evoked potentials could not account for the presence of half-harmonic synchronisation as measured by the PLV (compare Fig. 5C2 and D2 to C1 and D1).

**Fig. 6:**
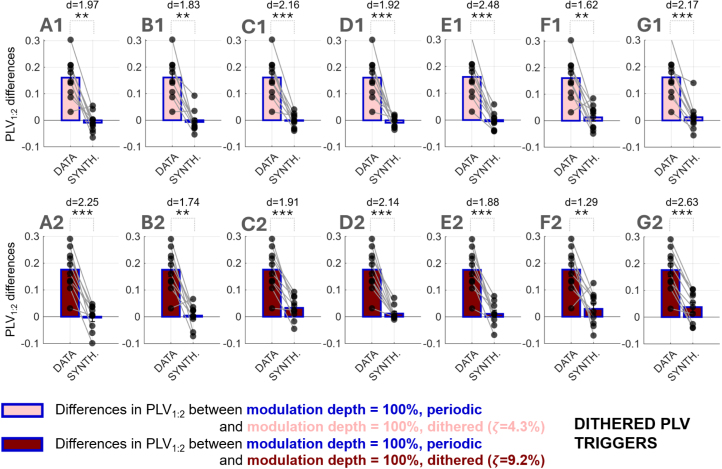
**Large discrepancy in**${\text{PLV}}_{\text{1:2}}$**reduction by dithering between empirical data and synthetic data generated according to the superposition of evoked potentials hypothesis.** The first row corresponds to differences in ${\text{PLV}}_{\text{1:2}}$ between the periodic condition and the condition with the lowest dithering level, and the second row corresponds to differences in ${\text{PLV}}_{\text{1:2}}$ between the periodic condition and the condition with the highest dithering level. Dithered PLV triggers are used in all cases. Columns correspond to different types of synthetic data generated according to the superposition hypothesis, all with optimised scale factor S and modulation factor $m_{\text{1:2}}$. **A:** Synthetic data using averaged evoked potentials; **B:** using averaged evoked potentials with components at half the stimulation frequency; **C:** using evoked potentials sampled on a single trial, single pulse basis; **D:** using evoked potentials with a linear modulation over time; **E:** using evoked potentials with a slow sinusoidal modulation over time; **F:** using the averaged flash VEP recorded in one participant; **G:** using flash VEPs from one participant sampled on a single trial, single pulse basis. Error bars represent the standard error of the mean. ** and *** indicate p≤ 0.01 and p≤ 0.001, respectively. Cohen’s effects sizes (d) are indicated above each plot.

Beyond linear superposition, evoked potentials could be modulated by non-linear sensory mechanisms such as saturation or gain control [32], [33]. To account for this, we additionally modulated evoked potentials at half the stimulation frequency. Such synthetic data could reproduce the response observed for the periodic condition with full modulation depth, but not for the dithered conditions (Fig. 5C3 and D3). When using dithered PLV triggers, the discrepancy with the empirical data was striking for both dithering levels (compare Fig. 5D1 and D3). This was also the case when considering time-varying modulation of evoked potentials at half the stimulation frequency (Fig S.3 in the supplementary material). Similar results were obtained when using the averaged flash VEP recorded in one participant (Fig S.13 in the supplementary material), or averaged evoked potentials including frequency components at half the stimulation frequency (Fig S.14 in the supplementary material). Finally, similar results were also obtained when constructing synthetic data by randomly sampling single-pulse, single-trial EPs or single-pulse, single-trial flash VEPs (thus restoring within-trial as well as between-trial variability), see Fig S.4 in the supplementary material. Statistical analyses revealed that, for all variants of synthetic data generated according to the superposition hypothesis, the discrepancy in ${\text{PLV}}_{\text{1:2}}$ reduction by dithering between empirical data and synthetic data was significant with a large effect size (Fig. 6, dithered PLV triggers). Even when accounting for within-trial as well as between-trial variability through evoked response/VEP sampling, the discrepancy remained pronounced with Cohen’s d≥1.91 and p≤ 0.001 (see Fig. 6C, G).

We investigated the alternative hypothesis of subharmonic entrainment using an oscillator model. We simulated the sine circle map, which is the simplest model describing the influence of periodic stimulation on a single neural oscillator, with the addition of noise to the oscillator’s frequency to approximately match 1:2 synchronisation for periodic stimulation to the empirical data. The resulting ${\text{PLV}}_{\text{1:2}}$ for dithered stimulation are more consistent with the empirical data for both types of PLV triggers (Fig. 5C4–D4) than the ${\text{PLV}}_{\text{1:2}}$ based on the superposition hypothesis, in particular for dithered PLV triggers. As in the empirical data, there exists in the oscillator model a dithering level which suppresses half-harmonic synchronisation even when the PLV is computed using dithered triggers. This is a signature of entrainment and makes the superposition of evoked potentials hypothesis unlikely (where evoked responses are simply shifted according to the dithered stimulation timing).

## Discussion

In this study, we showed using photic stimulation and EEG recordings in healthy participants that slightly jittering stimulation pulses (dithered stimulation) suppresses half-harmonic synchronisation relative to perfectly periodic flicker at the individual half-harmonic optimum frequency (Fig. 4), as previously predicted by mathematical modelling [19]. Notably, dithered stimulation suppressed half-harmonic synchronisation more than synchronisation at the stimulation frequency (in particular when using a windowed measure of synchronisation, Fig. 4D). This was also the case for the periodic condition with reduced stimulation depth, as predicted by theory.

Additionally, we demonstrated by generating synthetic data that the half-harmonic responses observed in participants cannot be explained by the superposition of evoked responses, even when evoked responses are modulated at the half-harmonic frequency (Fig. 5C2-3, D2-3). The discrepancy with empirical data was the largest when the PLV was computed with dithered PLV triggers that follow dithered stimulation timing variability (Fig. 6). Instead, the half-harmonic responses of a minimal oscillator model receiving periodic and dithered stimulation better matched the group level data (Fig. 5C4, D4), suggesting the presence of half-harmonic entrainment.

Together, these findings are consistent with the view that half-harmonic EEG synchronisation during photic stimulation predominantly reflects half-harmonic entrainment rather than the summation of evoked responses, and that dithering, as well as reducing stimulation amplitude, effectively suppress subharmonic entrainment.

### Limitations

The sample size is limited (n = 10), in part due to the rejection of datasets with insufficient half-harmonic response to periodic stimulation. Nonetheless, the measured effect size is large (Cohen’s d for the difference between ${\text{PLV}}_{\text{1:2}}$ for periodic stimulation with full modulation depth and the first/second dithering level is 1.77/2.06, respectively). While dithering also significantly suppressed superharmonic synchronisation, and more so than synchronisation at the stimulation frequency (Fig S.12B1, C1 in the supplementary material, p-values in the caption), we could not conclude on the potential of dithering to suppress superharmonic entrainment because we could not distinguish these superharmonic responses from the superposition of evoked potentials (Fig S.12). Due to experimental time constraints, we were also unable to test stimulation conditions that may better preserve the 1:1 response, in particular lower dithering levels than ζ=4.3%, and the combination of low dithering levels with reduced modulation depth. Such stimulation conditions may be as good or better than the periodic condition with 66% modulation depth at suppressing 1:2 while preserving 1:1 synchronisation. Additionally, we have not assessed the effects of dithering over the course of long stimulation periods. Since dithering introduces a random component in the timing of stimulation pulses, the emergence of adaptation is less likely. In the case of visual stimulation for example, less habituation was found in response to arrhythmic flashes [36], and more generally stimulus predictability reduces primary visual cortex response [37]. This should however be confirmed in future work. Finally, to assess whether the half-harmonic data may be more consistent with entrainment, we intentionally simulated a minimal (phase-only) oscillator model. More realistic models (e.g. coupled oscillators or neural mass models [13], [27], [29]) may better reproduce the data.

### Factors contributing to half-harmonic responses

The detection of half-harmonic entrainment depends both on the neural circuit generating the response (specifically, the characteristics of its 1:2 Arnold tongue), and stimulation parameters (such as stimulation frequency, amplitude, and pulse shape). Here, participants with no detected half-harmonic responses may have been characterised by smaller 1:2 Arnold tongues, and would have required lower or higher stimulation intensities than the stimulation intensity used in our study, or a more precise frequency tuning (steps of 2 Hz were used in the initial frequency sweep). With photic stimulation, individual variability in the gain of the corresponding sensory pathway (including retinal responsiveness and thalamic relay gain) may have also played a role in the presence of half-harmonic responses. In our dataset, the presence of a half-harmonic response and study inclusion could not be predicted by demographic characteristics (age and gender) or $f_{\text{max}1:2}$, but a stronger response at the stimulation frequency was predictive in single-predictor models (see Section B in the supplementary material). This suggests that participants with stronger synchronisation at the stimulation frequency may be more likely to exhibit detectable half-harmonic responses, possibly because of greater gain in the corresponding sensory pathway. However, this exploratory result should be interpreted cautiously given the limited sample size.

While the prevalence of half-harmonic responses to DBS in PD patients is higher than in our study (subcortical half-harmonic responses reported in 78% of PD patients in one study [15] and cortical half-harmonic responses in 80% of PD patients in another study [17]), previous work in PD showed that 1:2 Arnold tongues can vary significantly between patients [13], and that half-harmonic responses are limited to a specific range of stimulation amplitude.

### Half-harmonic responses are consistent with entrainment, not evoked-potential superposition

The nature of the 1:1 response to rhythmic sensory stimulation such as photic stimulation is still debated, with some studies pointing to a simple superposition of evoked responses [20], [38], and others to neural entrainment and resonance of neural circuits [21], [22], [23], [24]. Half-harmonic responses have been reported in humans [6], [25], [26], [27], [28], [29] and animals [32], [39] in response to photic stimulation, however their underlying mechanism has received little attention. While we could not distinguish in our data 1:1 and superharmonic responses from a superposition of evoked responses, we provided evidence that half-harmonic responses are inconsistent with the superposition of evoked responses hypothesis and may represent neural entrainment. Patient-specific synthetic data generated according to the superposition of evoked responses hypothesis failed to account for any 1:2 synchronisation. When adding an explicit half-frequency modulation of evoked responses (which could be the result of non-linear sensory mechanisms), the synthetic data could match the ${\text{PLV}}_{\text{1:2}}$ in response to periodic stimulation yet could not reproduce the ${\text{PLV}}_{\text{1:2}}$ observed for dithered stimulation, in particular for dithered PLV triggers (Fig. 5 and Fig. 6). We also note that since the illumination and modulation depth were the same for the periodic and dithered stimulation used in this analysis and the pulse timing differences under the dithered conditions are minimal, it is unlikely that a potential non-linear sensory mechanism would behave differently under the periodic and dithered conditions. Moreover, supplementary analyses that (1) substituted the averaged evoked potential for a flash VEP measured separately, (2) constructed averaged responses that already contain half-frequency components from the data, (3) sampled evoked responses or VEPs on a single trial, single pulse basis to incorporate within- and between-trial variability, or (4) included slow variations of evoked response over time led to the same mismatch under dithering.

Conversely, the oscillator model reproduced the drop in ${\text{PLV}}_{\text{1:2}}$ as ζ increases for dithered PLV triggers. Similarly to the data, the oscillator’s half-harmonic responses to dithered stimulation were not tightly temporally locked to every other stimulation trigger as revealed by ${\text{PLV}}_{\text{1:2}}$ with dithered PLV triggers (dithering had no impact on ${\text{PLV}}_{\text{1:2}}$ in the superposition hypothesis even with $m_{\text{1:2}}\neq 0$, Fig. 5D3). Additionally, dithering was previously tested in a more biologically realistic neural network model [19], with results consistent with the sine circle map. The model included interconnected, heterogeneous units and intrinsic noise. As in the sine circle map, it was shown that there exists in this model a dithering level which suppresses half-harmonic synchronisation even when the PLV is computed using dithered triggers (which is the case in the data but not in the superposition of evoked potentials hypothesis). While the presence of an on-going oscillation is sometimes cited as a prerequisite for entrainment [40], [41], recent data shows that subharmonic entrainment does not necessarily require an on-going oscillation that can be measured in the absence of stimulation, even when using invasive recordings [17]. Such a pre-existing oscillation could facilitate entrainment [42], however unsynchronised oscillators with the capacity to synchronise may be sufficient [43].

Together, the evidence we present weigh against the superposition hypothesis for half-harmonic responses and favour a half-harmonic entrainment mechanism sensitive to the temporal statistics of the pulse train beyond simple locked responses.

### A simple design rule to quench harmonic synchronisation: add jitter

We developed dithered stimulation as a method to suppress sub- and superharmonic entrainment using mathematical modelling [19]. Here, we confirmed that adding a modest amount of jitter to the timing of stimulation pulses (ζ=4.3%) while keeping the overall energy delivered constant can effectively suppress subharmonic synchronisation in response to sensory stimulation in humans, while maintaining some level of 1:1 synchronisation. We tested dithering at the individual frequency leading to the maximum 1:2 power response, with stimulation frequency varying between 19 and 43 Hz across included participants. In our limited sample, there was no association between stimulation frequency and efficacy of dithering, suggesting that dithering may be effective across frequencies, at least within this range. However, because different frequency bands likely arise from neural circuits with different dynamics, they may differ in the amount of pulse-timing variability required to suppress half-harmonic responses. This should be investigated in future work. Another study provided evidence that Poisson stimulation trains prevent superharmonic responses during optogenetic excitatory stimulation in mice [44], with a broad low power 1:1 response likely due to the large pulse timing variability. In our study, we note that PSD peak width at the stimulation frequency increases with dithering (see Fig. 4A2), but stays relatively sharp for low dithering levels.

It was recently suggested that half-harmonic entrainment to brain stimulation can functionally disconnect neural oscillations [15], [17]. Neural oscillations are thought to support cognitive and sensorimotor function through phase-based coupling (such as phase–amplitude coupling), thereby coordinating different frequency bands and brain regions [45], [46]. Harmonic entrainment of a neural oscillation to an external drive (the stimulation) introduces a rigid phase structure in the oscillation, which is likely to disrupt its physiological coupling regime and could compromise function. Alternatively, the strengthening of a neural oscillation through harmonic entrainment could in and of itself lead to pathological effects. For example, excessive 1:2 entrainment of the pro-kinetic gamma rhythm in PD patients with DBS could trigger dyskinesia, although this is not always the case and may depend on the motor state [11], [12], [17]. Similarly, 40 Hz photic stimulation for Alzheimer’s disease [5], [6], [7], [8] could strengthen the beta rhythm through half-harmonic synchronisation in the motor network, which could give rise to anti-kinetic effects. In brain stimulation approaches for epilepsy, subharmonic entrainment of the slow rhythm associated with epileptic seizures should be avoided [18]. When subharmonic synchronisation could lead to deleterious effects, dithering offers a practical engineering principle to suppress it.

Given that dithering or other types of noisy pulse trains have been reported to suppress harmonic responses in minimal and more realistic computational models [19], in mice using optonogenetics [44], and in humans using photic stimulation (this study), the efficacy of dithering in suppressing harmonic responses may generalise to other stimulation modalities. Such modalities may include transcranial alternating current stimulation, which is known to entrain neural oscillations [47], and DBS, which was recently shown to entrain neural oscillations at the half-harmonic of the stimulation frequency [11], [12], [13]. Dithering is expected to be effective for all stimulation waveforms as its efficacy is tied to the timing of the stimulation trigger. There should exist a (possibly different) dithering level which suppresses subharmonic responses regardless of waveform shape. This should however be verified in future work. From a practical perspective, dithered stimulation can be implemented in neurostimulators with limited capabilities by toggling within a finite set of stimulation frequencies [19].

Half-harmonic synchronisation in response to DBS has gathered a significant amount of interest, in part because of the potential involvement of half-harmonic entrainment in the therapeutic effects of DBS in movement disorders [14], [15], [16]. Since dithered stimulation can modulate half-harmonic synchronisation, it could provide a means to causally investigate the therapeutic relevance of half-harmonic synchronisation. It is however unclear how patient groups may react to dithering compared to healthy participants, given pathological neural dynamics, medication intake, and compensatory mechanisms. While previous theoretical work suggests that a suitable level of dithering should suppress the half-harmonic response in any time-invariant system displaying entrainment [19], compensatory mechanisms on various timescales could affect the efficacy the dithering. The efficacy of dithering in patient groups should therefore be investigated in future work. This will involve finding the lowest dithering level that sufficiently suppresses the half-harmonic response, for example using an incremental titration approach where the dithering level is increased in small steps starting from 0%, with ${\text{PLV}}_{\text{1:2}}$ as a readout. The duration required to reliably assess ${\text{PLV}}_{\text{1:2}}$ will depend on the variability and transients in the response of the relevant neural circuits as well as the level of noise in the measured signals. Optimal dithering levels may be different for different clinical populations, and could also vary between individuals.

### Dithering vs reducing stimulation amplitude

Theory also predicts that sufficiently reducing stimulation amplitude (which corresponds to modulation depth in this study) will suppress 1:2 entrainment, and this was confirmed by our results. When considering the global PLV, the periodic condition with reduced modulation depth suppressed half-harmonic synchronisation similarly to the intermediate dithering level, but better preserved synchronisation at the stimulation frequency. However, results were comparable when considering the windowed PLV. Reducing stimulation amplitude impacts 1:2 entrainment through a different mechanism than dithering: the system simply leaves the 1:2 Arnold tongue (see Fig. 1B1). The efficacy of a reduced stimulation amplitude in suppressing 1:2 synchronisation strongly depends on the system of interest (which determines the shape of the Arnold tongues) and stimulation parameters (where the system is with respect to the boundaries of the 1:2 and 1:1 Arnold tongues).

Whether reducing stimulation amplitude is a better choice than dithering to suppress 1:2 synchronisation while preserving 1:1 synchronisation will therefore depend on the system of interest. In some cases, it may be optimal to combine dithering with a reduced stimulation amplitude. Reducing stimulation amplitude may not always be possible, for example when supra-threshold stimulation is needed, when a certain amount of energy needs to be provided for therapeutic effect, or due to neurostimulator limitations. In such cases, manipulating the timing of stimulation pulses, as with dithering, would be the only way to suppress harmonic synchronisation. On the other hand, if stimulation amplitude can be reduced, it may be worth testing dithering, reduced stimulation amplitude, and a combination of both approaches. It should be noted that when precise pulse timing is critical for therapeutic efficacy, such as with phase-locked stimulation, dithering could significantly reduce therapeutic efficacy if the dithering level is too high compared to the required phase specificity.

### Conclusion

In summary, we demonstrated that dithering can suppress half-harmonic synchronisation to photic stimulation in humans. Moreover, we provided evidence that these half-harmonic responses are consistent with entrainment but not with the evoked potential superposition hypothesis. When stimulation amplitude cannot be reduced, these findings support dithering as a simple open-loop design principle for selective entrainment, and highlight its potential to enable brain stimulation therapies where there are physiological rhythms to reinforce and pathological rhythms that should not be entrained. Dithering could also enable new mechanistic investigations through its ability to modulate 1:2 synchronisation while ensuring the delivered energy remains constant.

## CRediT authorship contribution statement

**Benoit Duchet:** Writing – review & editing, Writing – original draft, Supervision, Methodology, Investigation, Funding acquisition, Formal analysis, Conceptualization. **Samini Subramaniam:** Writing – review & editing, Investigation. **Alexander Greenway:** Writing – review & editing, Investigation. **Shenghong He:** Writing – review & editing, Methodology. **Nicholas Shackle:** Writing – review & editing, Methodology. **Alek Pogosyan:** Writing – review & editing, Methodology. **Timothy Denison:** Writing – review & editing, Conceptualization. **Andrew Sharott:** Writing – review & editing, Resources. **Huiling Tan:** Writing – review & editing, Resources. **Rafal Bogacz:** Writing – review & editing, Conceptualization.

## Funding information

B.D. was jointly supported by the Royal Academy of Engineering and Rosetrees under the Research Fellowship programme. R.B. was supported by Medical Research Council, UK grant MC_UU_00003/1 and UKRI/MR/B000936/1. S.H., A.P., and H.T. were supported by the Medical Research Council, UK (MC_UU_00003/2), the Medical and Life Sciences Translational Fund (MLSTF) from the 10.13039/501100000769University of Oxford, United Kingdom, the National Institute for Health and Care Research (NIHR) Oxford Biomedical Research Centre, and the Rosetrees Trust, UK. S.H. was also supported by a Non-Clinical Postdoctoral Fellowship from the Guarantors of Brain, an International Exchanges Award (IES\R3\213,123) from The Royal Society, and a Senior Research Fellowship from 10.13039/501100000304Parkinson’s UK . A.S. and N.S. were supported by the 10.13039/501100000265Medical Research Council, UK grant MC_UU_00003/6. T.D. was supported by the Royal Academy of Engineering and the NIHR Invention for Innovation Programme.

## Declaration of competing interest

The authors declare the following financial interests/personal relationships which may be considered as potential competing interests: BD, TD, and RB are stakeholders in an intellectual property application based on this work. TD consult for and receive occasional honoraria from medical device companies, which produce devices used for Deep Brain Stimulation. TD is a stakeholder and a Director of Amber therapeutics.

## Data Availability

The data collected as part of this work are openly available at https://doi.org/10.60964/rnd-e9mr-v731.

## Supplementary data

MMC S1 Supplementary text, tables, and figures. MMC S1
