# Supplementary material for: Dithering suppresses half-harmonic neural synchronisation to photic stimulation in humans
Source: Brain Stimul. 2026 May-Jun;19(3):None. doi: 10.1016/j.brs.2026.103111 (PMC13328066; doi:10.1016/j.brs.2026.103111)
Supplement: MMC S1 — Supplementary text, tables, and figures. [file mmc1.pdf]

Supplementary material

A Additional synthetic data with superposition of evoked potentials

To assess whether the PLVs observed in data at the 1:2 subharmonic could be accounted for simply by sensory evoked potentials, we also generated synthetic data using two other types of averaged evoked potentials.

In figure S.13, we used the averaged flash VEP obtained by averaging the EEG response to 45 flashes (two-second interval between flashes) at 100% modulation depth in one participant (participant 10). This was done independently for each EEG channel, and the EEG was band-pass filtered between 1 and 45 Hz before averaging. This averaged flash VEP was truncated at 400 ms, and was used for all participants. The scale factor S, and when applicable the modulation factor $m_{1:2}$, were determined independently for each subject as before.

In figure S.14, we used averaged evoked potentials including frequency components at half the stimulation frequency. These averaged evoked potentials were obtained by averaging $2/f_{\text{stim}}$ long epochs directly following every other stimulation triggers in trials at the stimulation frequency considered, with $\zeta = 0\%$, and a modulation depth of 100%. This was done independently for each EEG channel and each participant, using the corresponding EEG data high-passed at 1 Hz, and low-passed at 80 Hz, with a notch filter at 50 Hz. The scale factor S, and when applicable the modulation factor $m_{1:2}$, were determined independently for each subject as before. In the perfectly periodic case and with $m_{1:2} = 0$, frequency components in the averaged evoked potential at half the stimulation frequency cancel out. However this is in general not the case with dithering, and this experiment allowed us to investigate whether interactions between $m_{1:2} \neq 0$ and frequency components in the averaged evoked potential at half the stimulation frequency might give rise to the PLV pattern observed in the data with dithered stimulation.

B Predicting half-harmonic response to periodic stimulation

Six out of 16 participants were not included in the analysis due to insufficient half-harmonic response to periodic stimulation. We investigated whether participant's

half-harmonic responses and study inclusion could be predicted by demographic (age and gender) or electrophysiological characteristics ($f_{\text{max }1:2}$ and $\text{PLV}_{1:1}$). Using multivariate linear regression, none of the predictors were found to be significantly associated with $\text{PLV}_{1:2}^{\text{win}}$ (age: p = 0.48, gender: p = 0.44, $f_{\text{max }1:2}$: p = 0.77, and $\text{PLV}_{1:1}$: p = 0.17). Using multivariate logistic regression, none of the predictors were found to be significantly associated with the binary outcome of inclusion in the analysis (age: p = 0.29, gender: p = 0.38, $f_{\text{max }1:2}$: p = 0.41, and $\text{PLV}_{1:1}$: p = 0.14), which was determined by thresholding $\text{PLV}_{1:2}^{\text{win}}$. Since multivariate models may be underpowered given the small sample size (n = 15), we also considered single-predictor logistic regression models. Only $\text{PLV}_{1:1}$ was significantly associated with inclusion in the study (p = 0.035).

C Trial rejection rates

The average percentages of rejected trials across all included participants was similarly low across conditions but not identical. The periodic condition with 66% modulation depth had 7.5% of rejected trials, the periodic condition with 100% modulation depth had 15.0% of rejected trials, the dithered condition with $\zeta = 4.3\%$ had 12.5% of rejected trials, and the dithered condition with $\zeta = 9.2\%$ had 5.0% of rejected trials. To verify that asymmetric trial rejection across conditions did not bias our results, we repeated the main PLV analyses only including subjects with no trial rejections across all conditions (n = 6). The results are very similar to results including subjects with rejected trials (compare Fig S.11 and Fig 4C-D). While statistical significance for the intermediate dithering level is lost (comparison of ratios relative to the periodic condition with full modulation depth at 1:1 and 1:2), this intermediate dithering level still suppresses 1:2 synchronisation reliably (p = 0.016).

D Non-linear models of period-doubling in visual pathway

To illustrate that modulating the visual response gain alternatively by $1 + m_{1:2}$ and $1 - m_{1:2}$ is a general approach to introduce period-doubling, we simulated two models with relevance to a potential period-doubling in the visual pathway. In both cases, the resulting period doubling behavior of the model could be described by our approach after a short transient (Fig S.2).

First, we considered a non-linear saturation model given by

$$x_{n+1} = \text{Sat}(ax_n + U), \qquad \text{Sat}(x) = \begin{cases} x, & \text{if } x \in [0,1], \\ 0, & \text{otherwise,} \end{cases} \tag{S.1}$$

with x_n the response to flash n, the input strength $U = 1$ and $a = -2$.

Second, we considered a non-linear feedback model previously used to model period-doubling in the visual response gain of the salamander [32]. We assumed that

the response to flash n was given by $x_n = C\,g(y_n)$ using the gain function

$$g(y) = \frac{1}{1+y^4}, \tag{S.2}$$

and the feedback variable y_n satisfying

$$y_{n+1} = e^{-\frac{1}{f\tau}}\Big(BC\,g(y_n) + y_n\Big). \tag{S.3}$$

We used $f = 32$ Hz, $\tau = 15$ ms, $B = 35$, and $C = 1$.

E Supplementary tables

Inclusion criteria	• Participant is willing and able to give informed consent for participation in the study<br>• Participant is mobile and can come to the lab without assistance<br>• Right-handed<br>• 20 to 60 years old<br>• Fluent in English<br>• Normal or corrected to normal vision<br>• No current significant medical condition<br>• No personal and/or family history of epilepsy<br>• Not currently taking any medications (except the contraceptive pill)<br>• Not pregnant or planning to get pregnant for the duration of the study
Exclusion criteria	• History of neurological conditions or currently diagnosed with a neurological condition (including paralysis, muscle weakness, poor coordination, loss of sensation, seizures, confusion, pain, and altered levels of consciousness)<br>• Any kind of epilepsy (including photosensitive epilepsy), or a family history of any kind of epilepsy.<br>• Any history or family history of seizures (including febrile convulsions in childhood)<br>• Any known significant sensitivity to flashing lights, or previous adverse experience in response to flashes, alternating patterns, or stripes of contrasting colours (for example from television or computer screen, strobe lights, flickering natural light such as sunlight flickering through trees...)<br>• History of migraines<br>• History of psychiatric conditions or currently diagnosed with a psychiatric condition, including bipolar disorder, depression or anxiety disorders<br>• Current consumption of any medication (except the contraceptive pill), including psychotropic drugs (such as antidepressants and neuroleptics)<br>• Eye disease (including glaucoma, retinitis, retinopathy, or macular degeneration)<br>• Any cardiac pathology<br>• Planning to get pregnant or likely to be pregnant during the course of the study<br>• Participant slept at least one hour less than their normal sleeping duration before the experimental session.<br>• Consumption of 3 or more units of alcohol in the last 24 hours<br>• Withdrawal from regular alcohol consumption<br>• High consumption of caffeine (more than one cup of coffee, or other source of caffeine in the last hour)<br>• Consumption of recreational drugs in the last 24 hours

Table A: Inclusion and exclusion criteria used in the study.

	periodic, mod. depth = 66%	periodic, mod. depth = 100%	$\zeta = 4.3\%$, mod. depth = 100%	$\zeta = 9.2\%$, mod. depth = 100%	average across conditions
within-subject SD (1:1)	0.0498	0.0654	0.0614	0.0442	0.0552
between-subject SD (1:1)	0.1639	0.1396	0.0556	0.0268	0.0965
within-subject SD (1:2)	0.0489	0.0645	0.0612	0.0439	0.0546
between-subject SD (1:2)	0.1633	0.1397	0.0554	0.0266	0.0962

Table B: Within- and between-subject variability in PLV 1:1 and PLV 1:2. For each condition, the within-subject standard deviation (SD) was obtained as the subject-level SD across repeats, averaged across subjects. For each condition, the between-subject SD was obtained as the SD across subjects of subject-level repeat averages.

F Supplementary figures

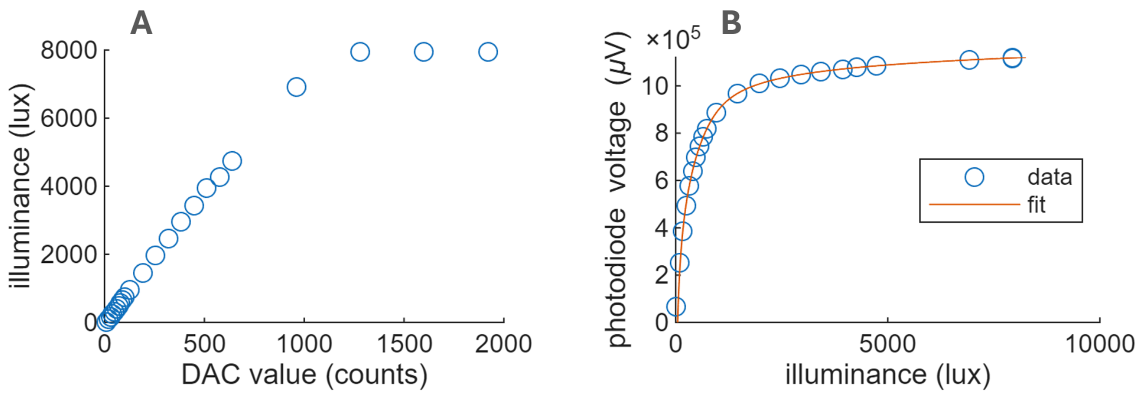

Figure S.1: LED panel and photodiode calibration. **A**: Illuminance measured at the LED panel's diffuser as a function of the digital-to-analog converter (DAC) value set in the microcontroller. This curve characterises the LED panel's response. The maximum DAC value used in this study is 1100 counts (just at the onset of saturation). **B**: Voltage measured by the photodiode at the LED panel's diffuser as a function of illuminance, also measured at the LED panel's diffuser. This curve characterises the photodiode's response. Data points are shown as blue circles, and the bi-exponential fit used to correct the output of the photodiode is shown in red. Illuminance was measured using a light meter (RS-92 Light Meter, RS PRO).

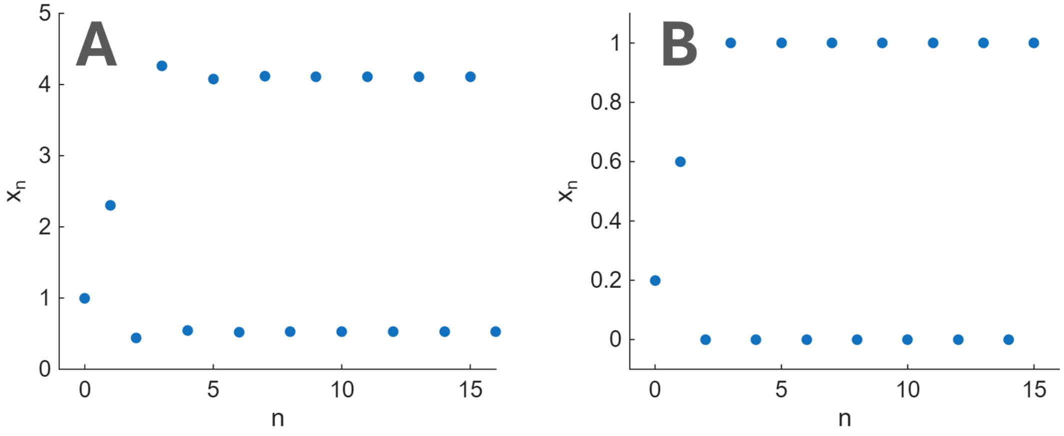

Figure S.2: Modulation of flash response at the half-harmonic frequency in two non-linear models. The response to flash n is modulated by x_n. **A**: Response of a non-linear feedback model. **B**: Response of a saturation model. Model details are given in Section D.

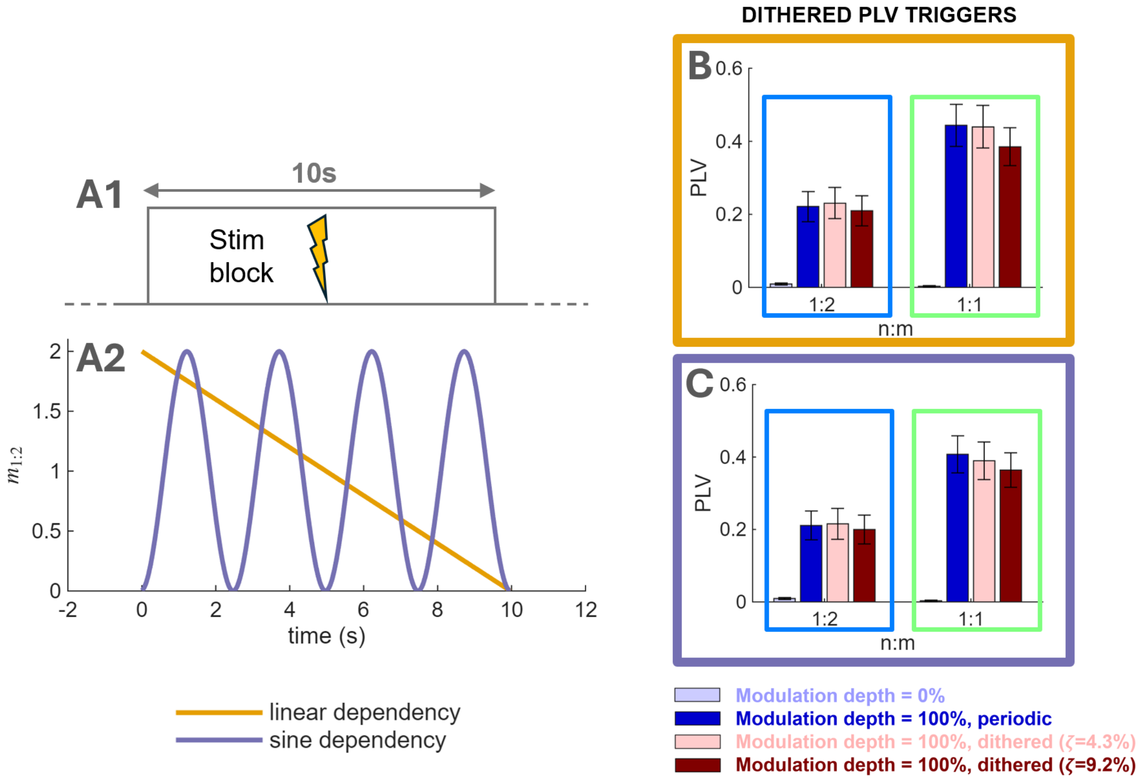

Figure S.3: Even with a time-dependent $m_{1:2}$, half-harmonic responses to photic stimulation are inconsistent with the superposition of evoked potentials. Synthetic data were generated as in Fig 5D3, but with $m_{1:2}$ varying throughout each stimulation block as shown in A1-2. The PLV at the stimulation frequency (1:1) and its half-harmonic (1:2) at the group level (based on dithered PLV triggers) are shown for a linear and sine time-dependency of $m_{1:2}$ in panels B and C, respectively. Error bars represent the standard error of the mean.

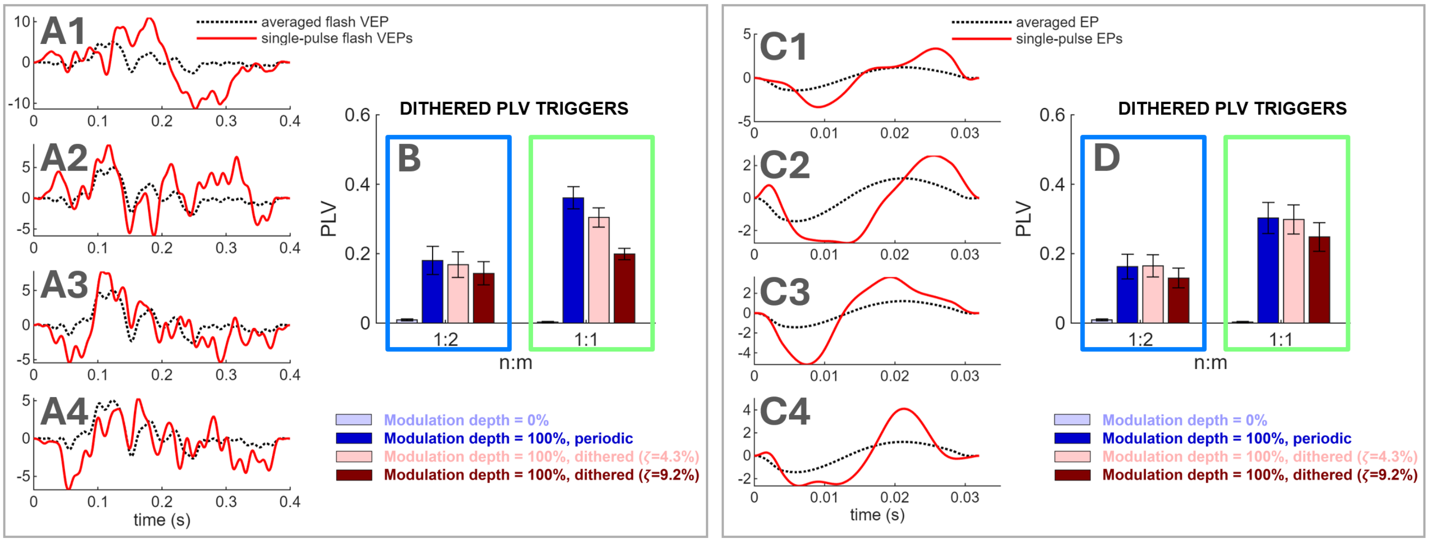

Figure S.4: Even when sampling single-pulse evoked responses/flash VEPs, half-harmonic responses to photic stimulation are inconsistent with the superposition of evoked potentials. Synthetic data were generated as in Fig S.13C3, but by sampling single-pulse, single-trial flash VEPs (examples are shown in A1-A4) rather than using the averaged flash VEP. The PLV at the stimulation frequency (1:1) and its half-harmonic (1:2) at the group level (based on dithered PLV triggers) are shown for the resulting synthetic data in B. Similarly, synthetic data were generated as in Fig 5D3, but by sampling single-pulse, single-trial EPs (examples are shown in C1-C4) rather than using the averaged EP. The PLV at the stimulation frequency (1:1) and its half-harmonic (1:2) at the group level (based on PLV dithered triggers) are shown for the resulting synthetic data in D. Error bars represent the standard error of the mean.

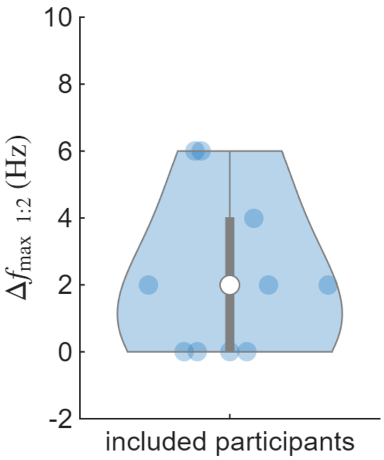

Figure S.5: Variability of $f_{\mathrm{max}\ 1:2}$ across trials in included participants.

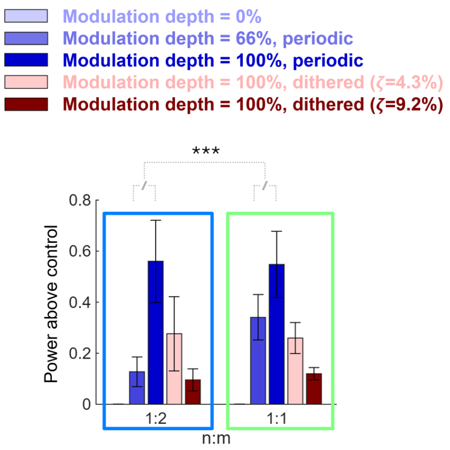

Figure S.6: Group level power above control at the stimulation frequency (1:1) and its half-harmonic (1:2) without normalisation. Error bars represent the standard error of the mean. *** indicates p≤0.001.

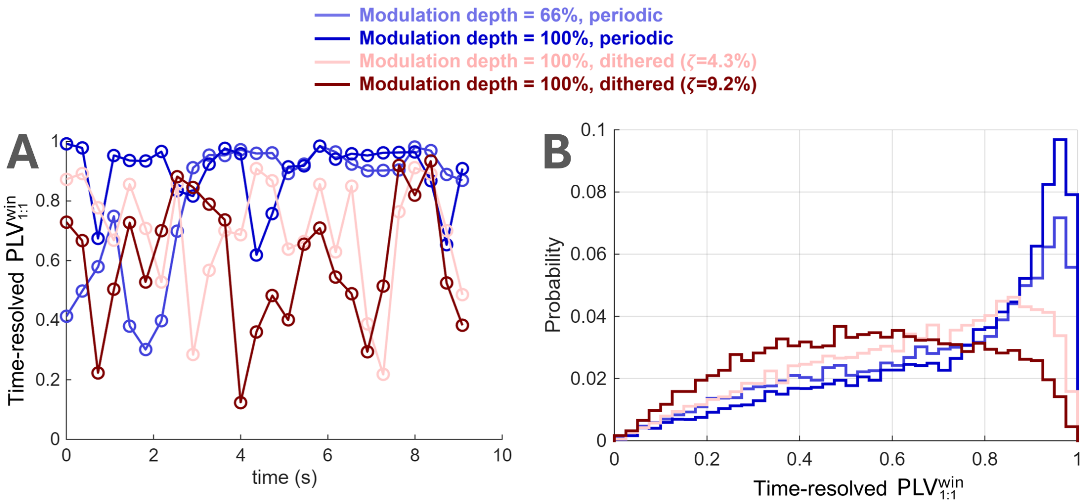

Figure S.7: The windowed PLV captures transient phase synchronisation. A: Example showing the (time-resolved) windowed PLV 1:1 over time for all conditions (Oz channel). The dithered conditions show periods of high phase synchronisation with periods of low synchronisation, which explains the difference between the global and averaged windowed PLV at the 1:1 ratio for these conditions. **B**: Probability density of (time-resolved) windowed PLV 1:1 values across participants and channels for all conditions, confirming this explanation at the group level.

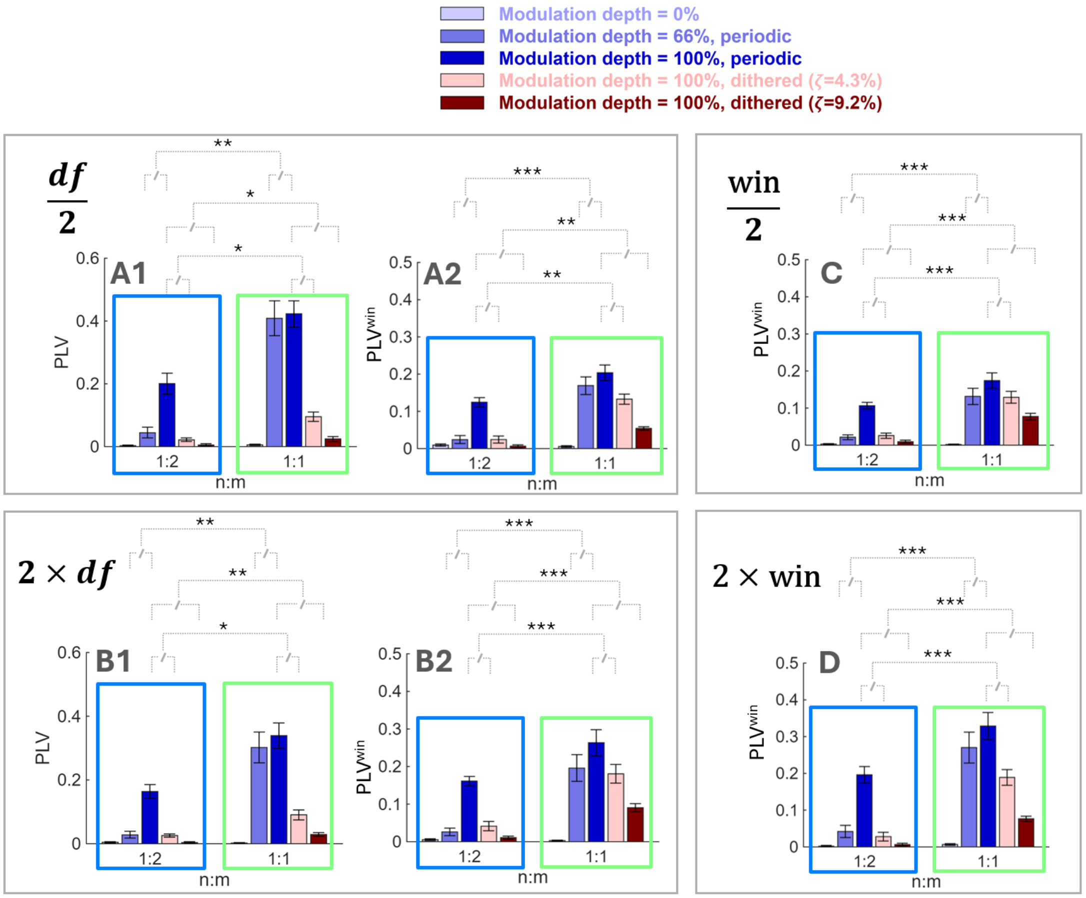

Figure S.8: Sensitivity analyses pertaining to filter half-width and PLV window duration. A, B: PLV analyses of healthy participant data were repeated using half, and twice the filter half-width *df* used in the main text, i.e. 0.5 Hz and 2 Hz for a 10 Hz signal, respectively. Global PLV results are shown in panels A1, and B1, and windowed PLV results in panels A2, B2. **C, D:** The windowed PLV analysis of healthy participant data was repeated using half, and twice the window duration used in the main text, i.e. 250 ms and 1s for a 40 Hz signal, respectively. Error bars represent the standard error of the mean. *, **, and *** indicate $p \leq 0.05$, $p \leq 0.01$, and $p \leq 0.001$, respectively.

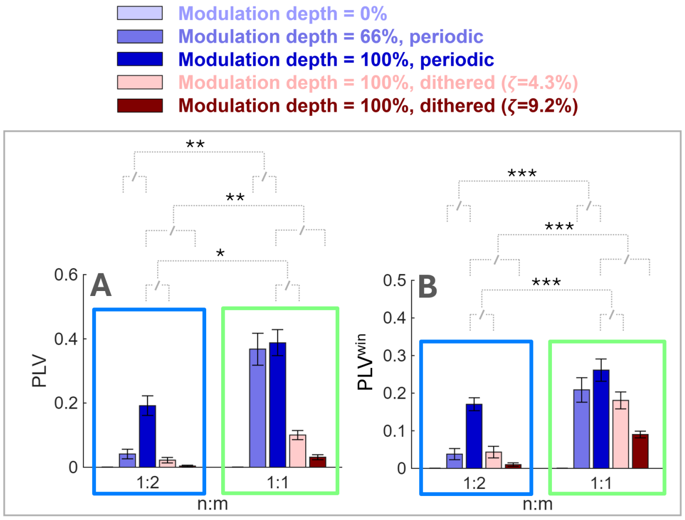

Figure S.9: Using control data rather than pink noise to remove the contribution of chance phase locking in PLV estimates. PLV analyses of healthy participant data were repeated using control data (modulation depth = 0%) instead of pink noise to remove the contribution of chance phase locking in PLV estimates. Global PLV results are shown in panel A, and windowed PLV results in panel B. Error bars represent the standard error of the mean. *, **, and *** indicate p≤0.05, p≤0.01, and p≤0.001, respectively.

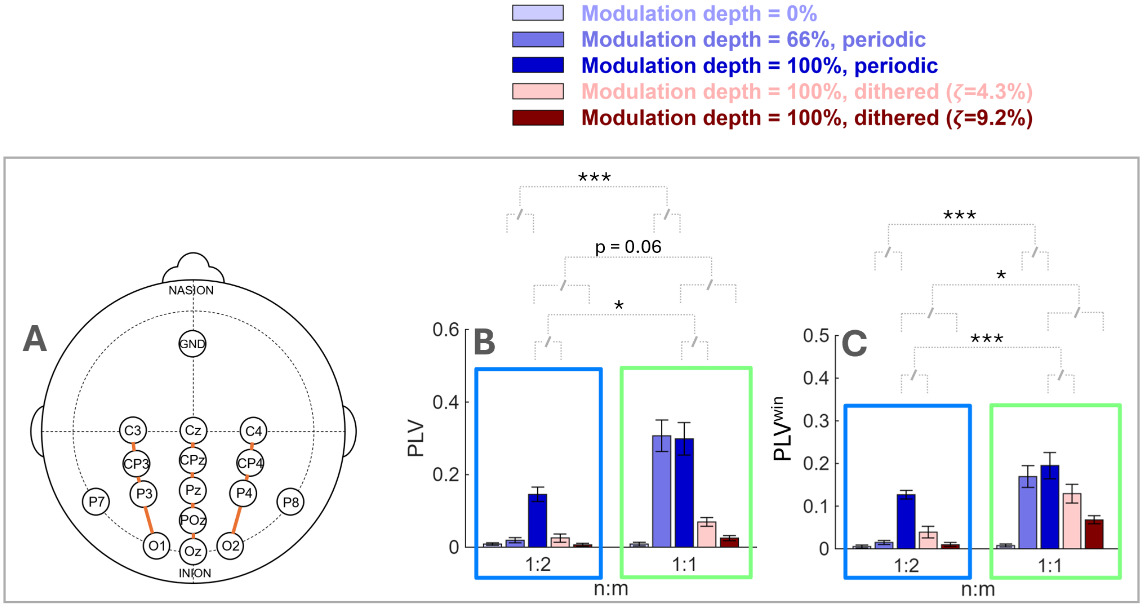

Figure S.10: Replication with a bipolar montage. PLV analyses of healthy participant data were repeated using the bipolar pairs depicted by oranges lines in panel A. Global PLV results are shown in panel B, and windowed PLV results in panel C. Error bars represent the standard error of the mean. *, and *** indicate p≤0.05, and p≤0.001, respectively.

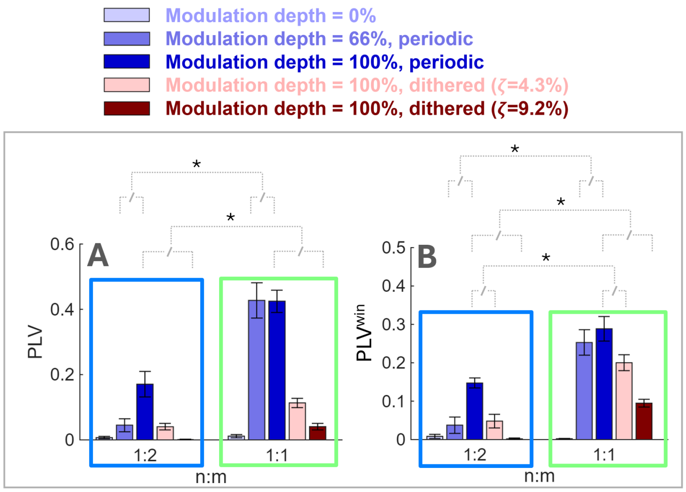

Figure S.11: Only including participants with no rejected trials. PLV analyses of healthy participant data were repeated only including participants with no rejected trials (n = 6). Global PLV results are shown in panel A, and windowed PLV results in panel B. Error bars represent the standard error of the mean. * indicates $p \leq 0.05$.

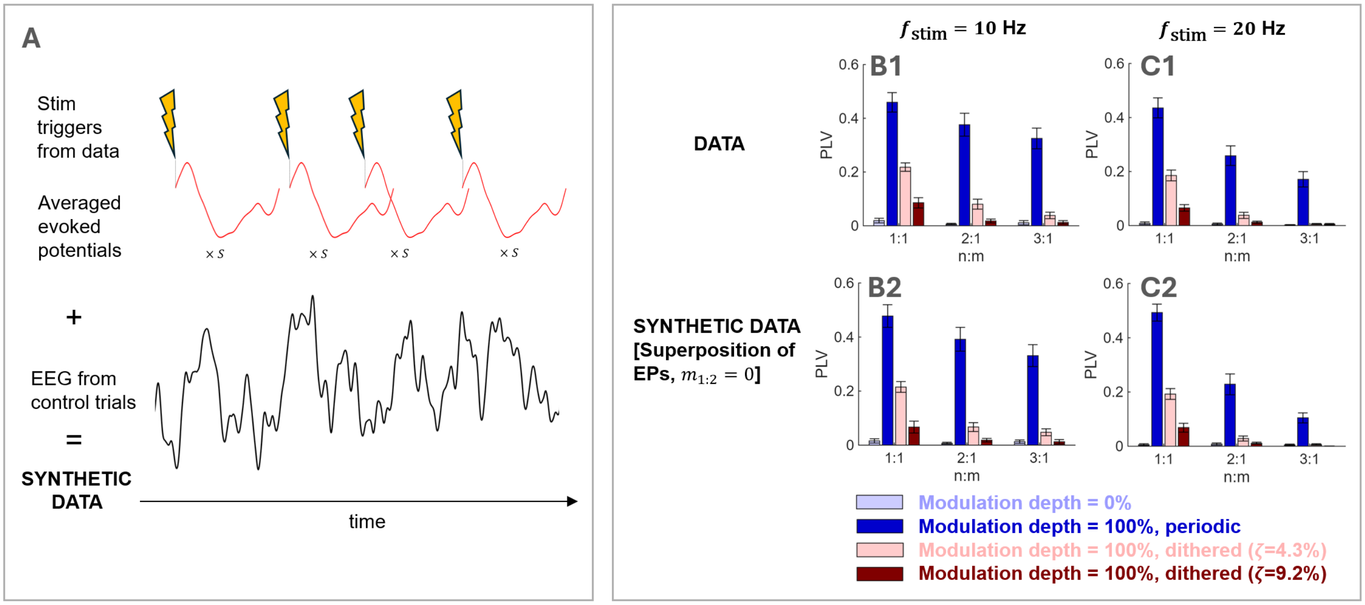

Figure S.12: Superharmonic responses to photic stimulation can be reproduced by the superposition of evoked responses. A: Schematic illustrating how synthetic data were generated according to the superposition of evoked potentials hypothesis. For each participant, the scale factor S was determined to match the data $PLV_{1:1}$ for the periodic condition. **B-C**: PLV at the stimulation frequency (1:1) and two superharmonic (2:1 and 3:1) at the group level, using fixed PLV triggers. Comparison between the empirical data and synthetic data generated according to the superposition of evoked potentials hypothesis. We note that superharmonic responses are strongly reduced by dithering in both cases. In the data (B1-C1), synchronisation was reduced at the superharmonics of stimulation more than at the stimulation frequency (comparison of ratios relative to the periodic condition with full modulation depth at 1:1 vs 2:1 and 3:1) For 10 Hz stimulation (B1): for 2:1 vs 1:1, $p = 0.001$ ($\zeta = 4.3\%$), and $p = 0.0039$ ($\zeta = 9.2\%$); for 3:1 vs 1:1, $p = 0.001$ ($\zeta = 4.3\%$), and $p = 0.0039$ ($\zeta = 9.2\%$). For 20 Hz stimulation (C1): for 2:1 vs 1:1, $p = 0.001$ ($\zeta = 4.3\%$), and $p = 0.002$ ($\zeta = 9.2\%$); for 3:1 vs 1:1, $p = 0.002$ ($\zeta = 4.3\%$), and $p = 0.0137$ ($\zeta = 9.2\%$). All these tests were one-tailed. Panels B-C share the same legend, and error bars represent the standard error of the mean. Note that these stimulation frequencies did not produce subharmonic responses to periodic stimulation with full modulation depth.

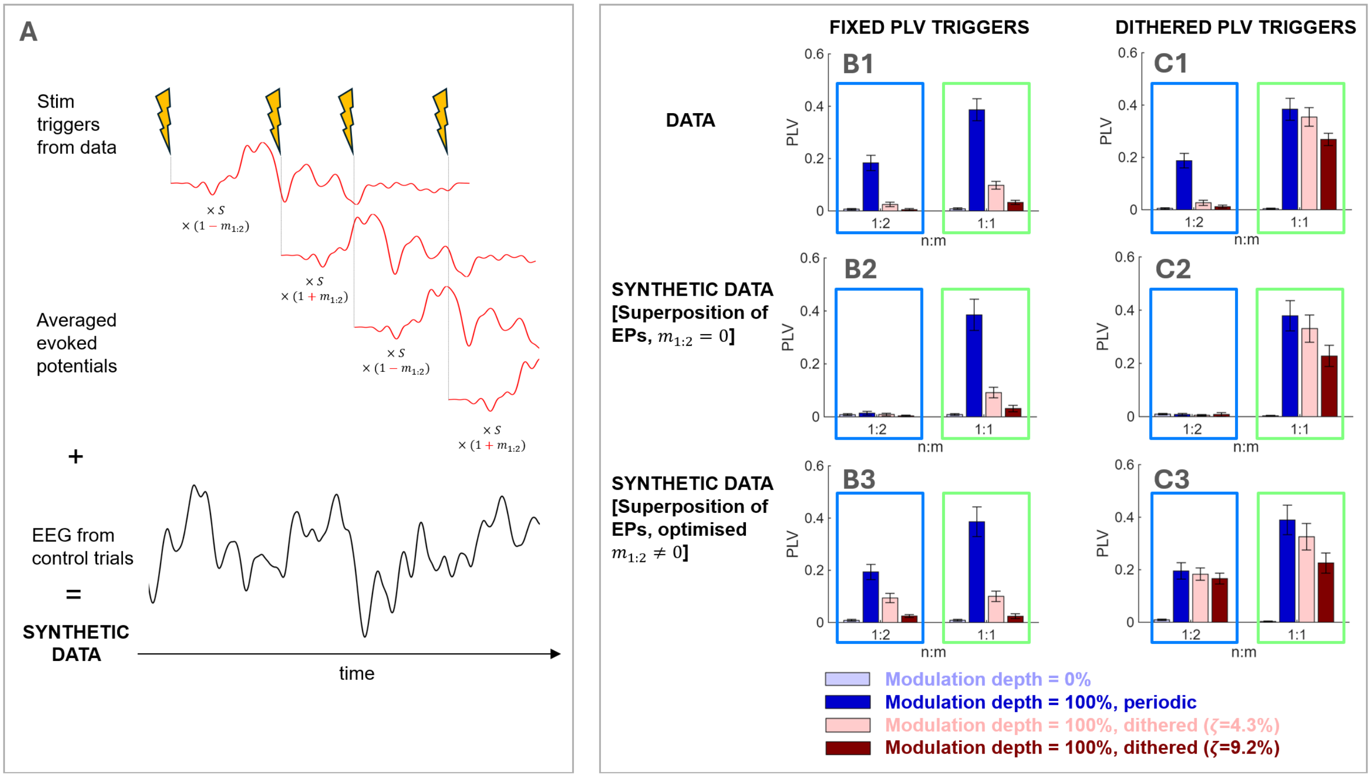

Figure S.13: Half-harmonic responses to photic stimulation are inconsistent with the superposition of flash VEPs. A: Schematic illustrating how synthetic data were generated according to the superposition of evoked potentials hypothesis, using the averaged flash VEP obtained in one participant. For each participant, the scale factor S was determined to match the data $\text{PLV}_{1:1}$ for the periodic condition. In B3 and C3, the modulation factor $m_{1:2}$ was chosen to match the data $\text{PLV}_{1:2}$ for the periodic condition for each participant. **B-C**: PLV at the stimulation frequency (1:1) and its half-harmonic (1:2) at the group level, for fixed PLV triggers (B) and dithered PLV triggers (C). Comparison between the empirical data and synthetic data generated according to the superposition of evoked potentials hypothesis (without and with modulation at the half-frequency). Panels B-C share the same legend, and error bars represent the standard error of the mean.

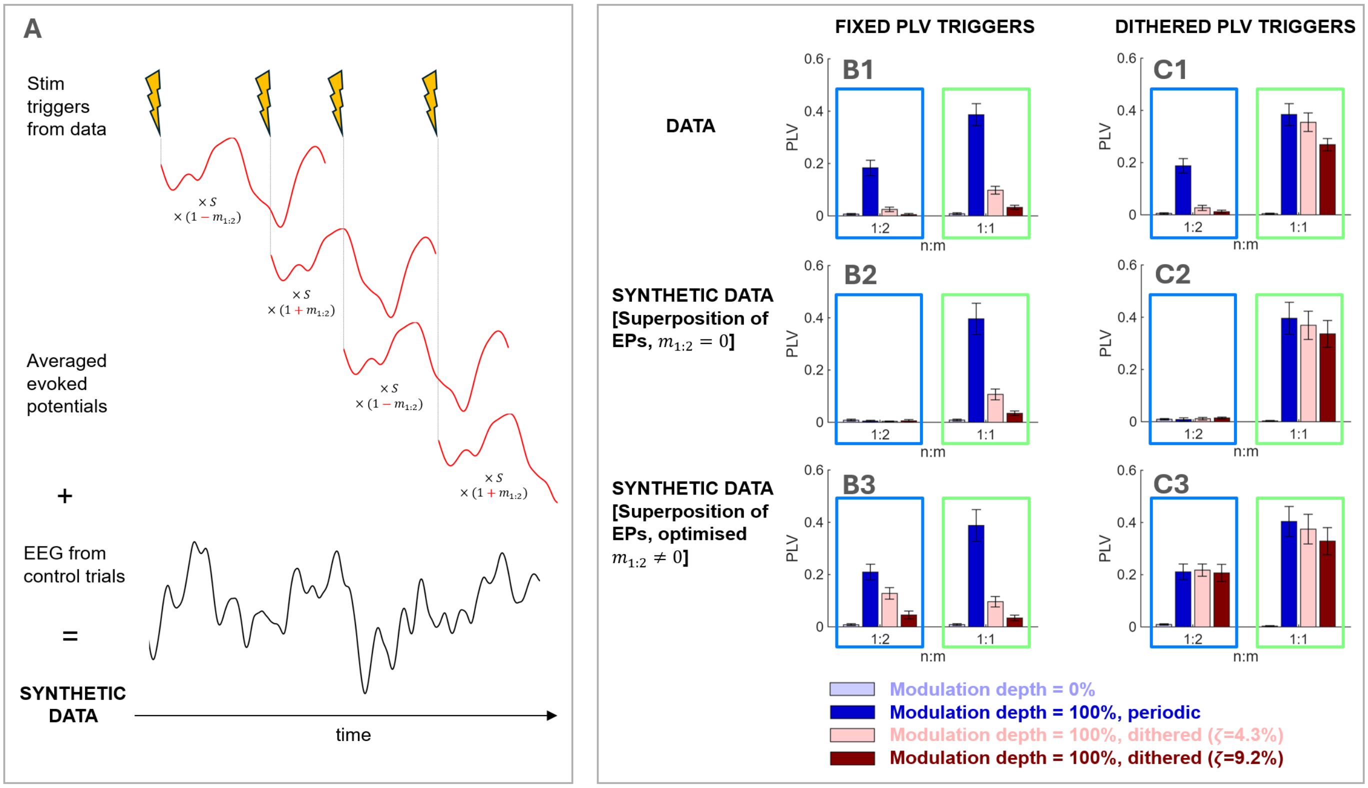

Figure S.14: Half-harmonic responses to photic stimulation are inconsistent with the superposition of evoked potentials including frequency components at half the stimulation frequency A: Schematic illustrating how synthetic data were generated according to the superposition of evoked potentials hypothesis, even when using averaged evoked potentials including frequency components at half the stimulation frequency. For each participant, the scale factor S was determined to match the data $\text{PLV}_{1:1}$ for the periodic condition. In B3 and C3, the modulation factor $m_{1:2}$ was chosen to match the data $\text{PLV}_{1:2}$ for the periodic condition for each participant. **B-C**: PLV at the stimulation frequency (1:1) and its half-harmonic (1:2) at the group level, for fixed PLV triggers (B) and dithered PLV triggers (C). Comparison between the empirical data and synthetic data generated according to the superposition of evoked potentials hypothesis (without and with modulation at the half-frequency). Panels B-C share the same legend, and error bars represent the standard error of the mean.
